# Supplementary material for: Voluntary Wheel Running Mitigates Disease in an Orai1 Gain-of-Function Mouse Model of Tubular Aggregate Myopathy
Source: Cells. 2025 Sep 4;14(17):1383. doi: 10.3390/cells14171383 (PMC12427812; doi:10.3390/cells14171383)
Supplement: Supplementary file 1 [file cells-14-01383-s001.zip › GS VWR Figures 20250821.pptx]

## Slide 1
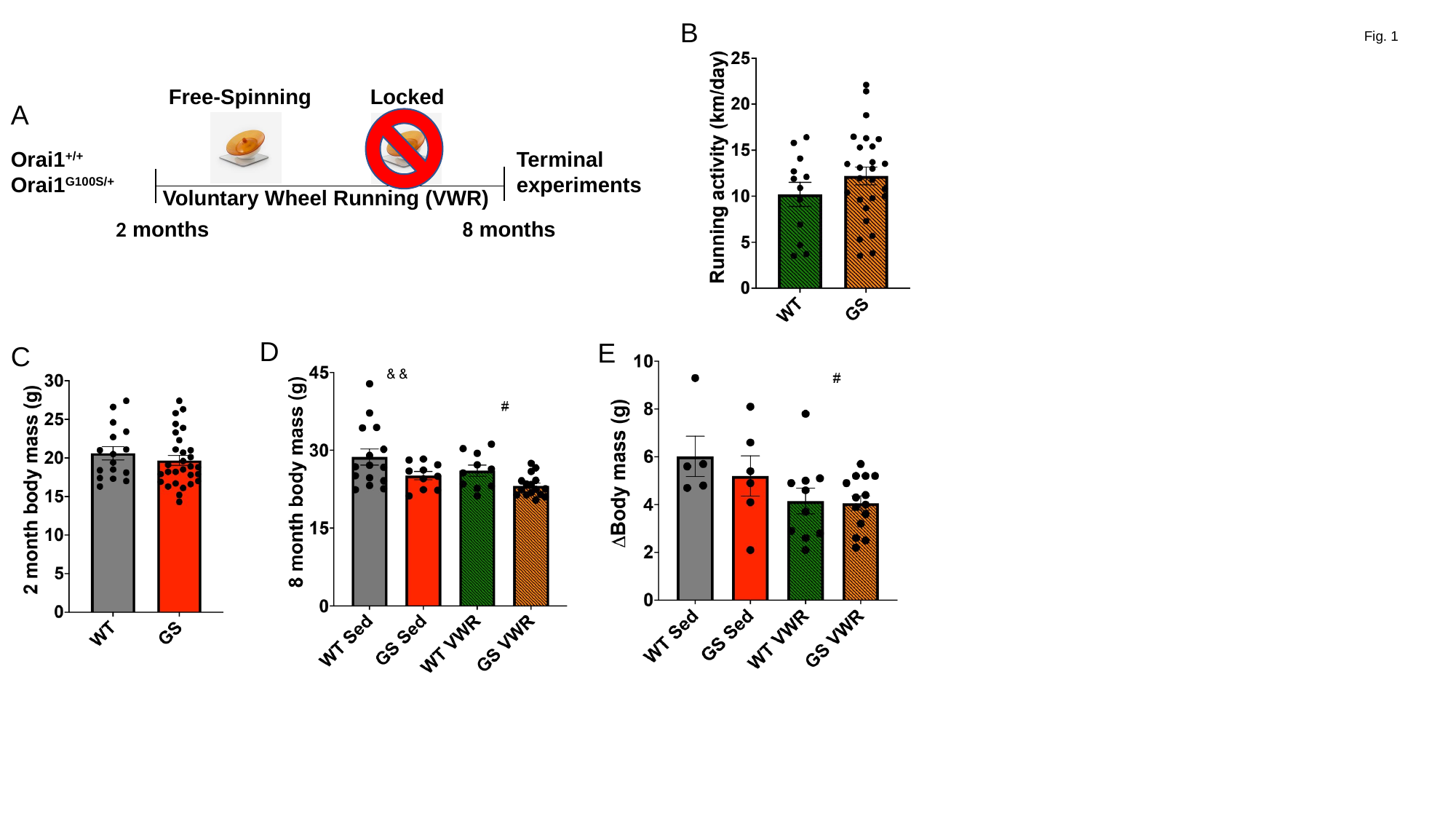

B
# Fig. 1
Free-Spinning
Locked
A
Orai1+/+
Orai1G100S/+
Terminal experiments
Voluntary Wheel Running (VWR)
2 months
8 months
D
E
C

## Slide 2
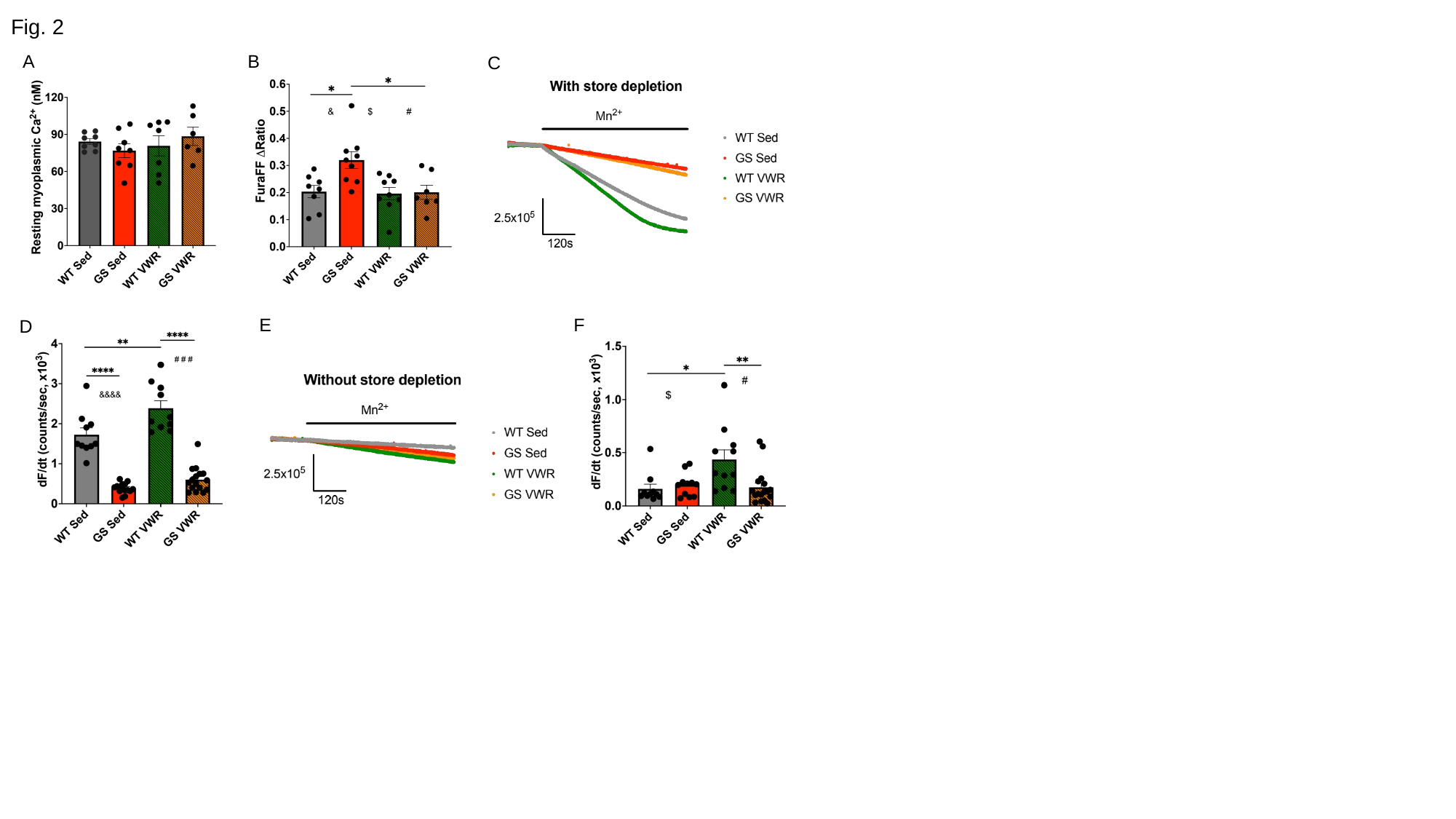

# Fig. 2
A
B
C
E
F
D

## Slide 3
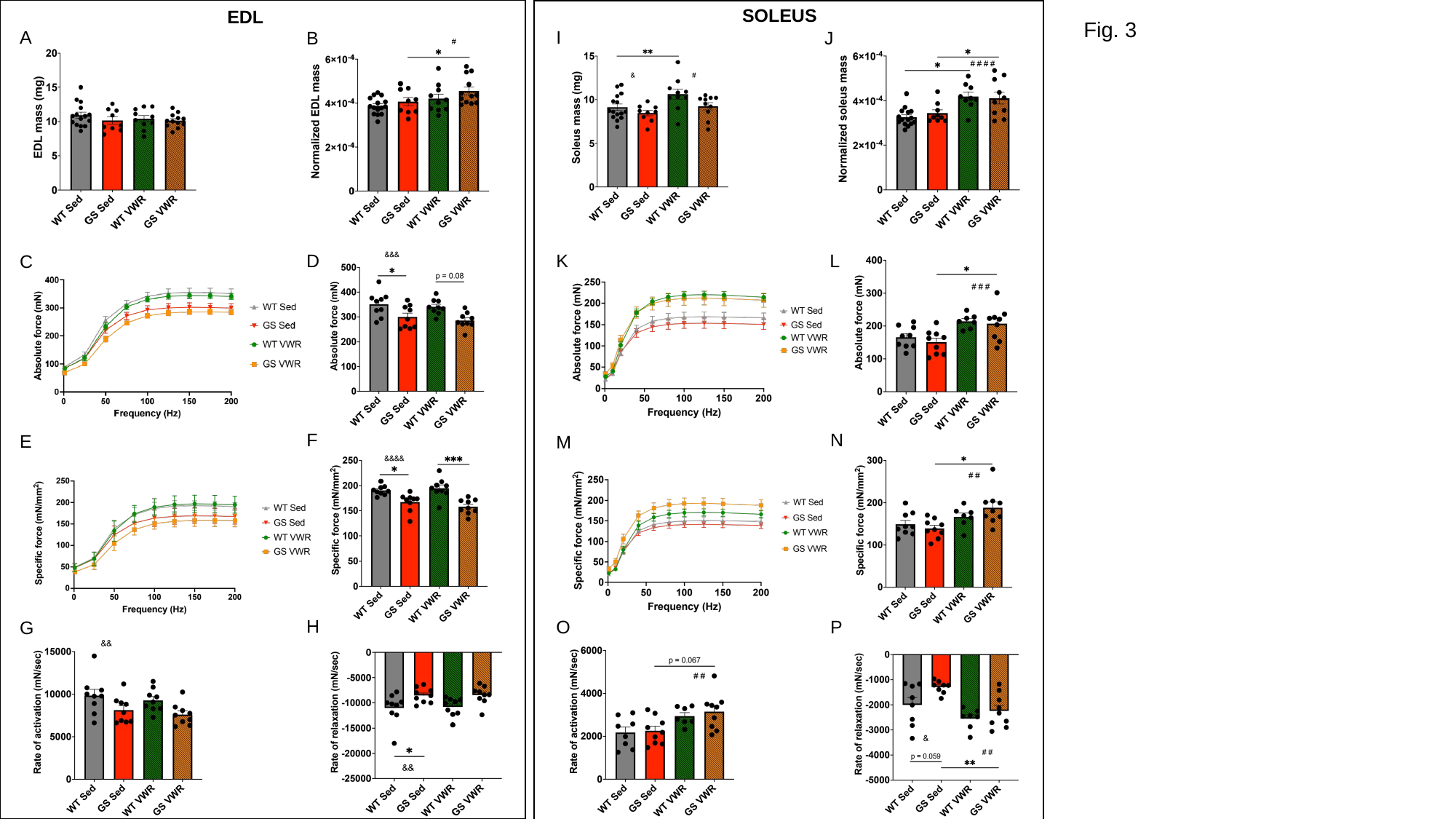

SOLEUS
EDL
Fig. 3
A
I
J
B
K
L
D
C
F
N
E
M
H
O
P
G

## Slide 4
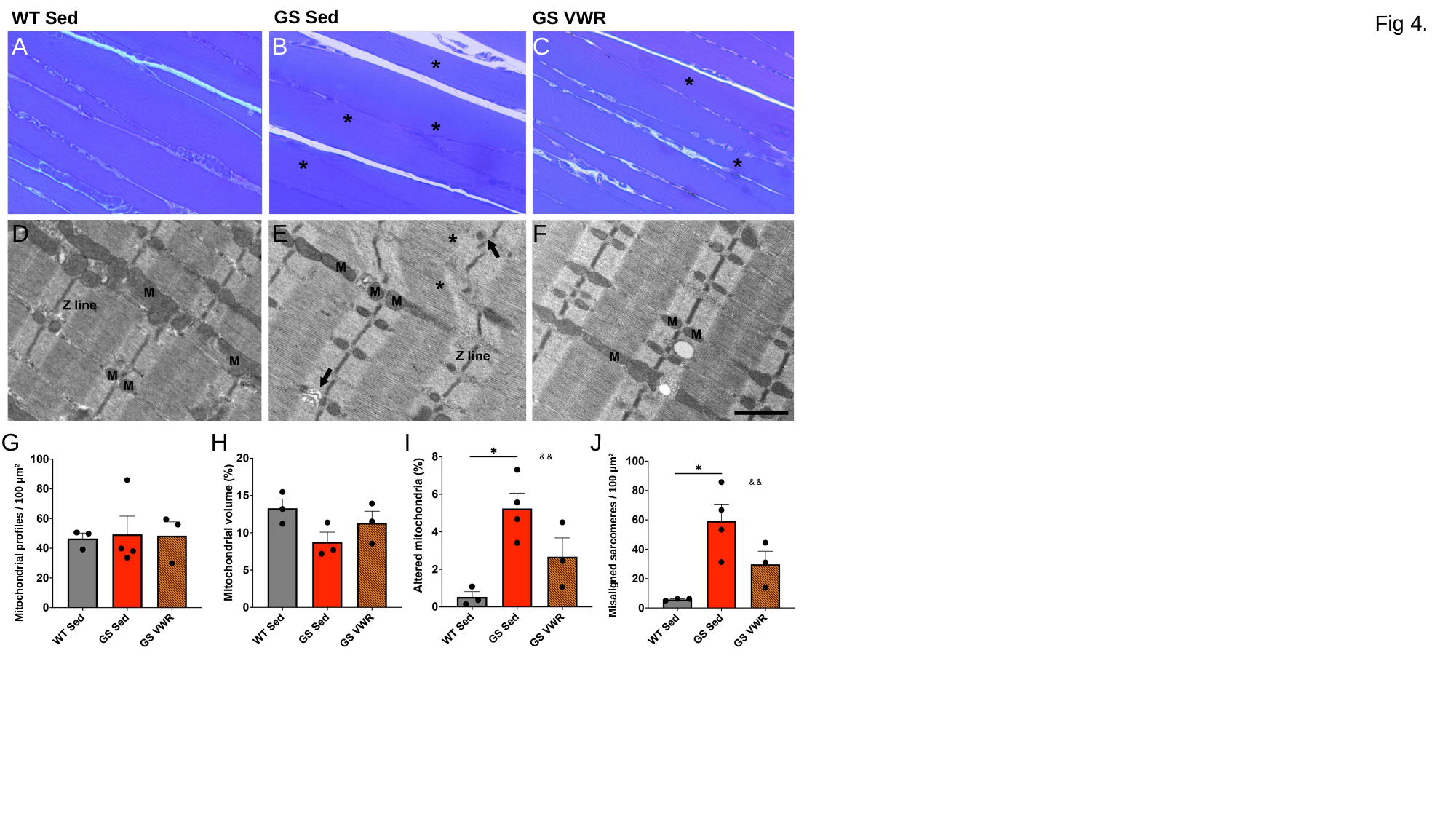

GS Sed
WT Sed
GS VWR
# Fig 4.
A
B
C
D
E
F
G
H
I
J
Misaligned sarcomeres / 100 μm2
Mitochondrial profiles / 100 μm2

## Slide 5
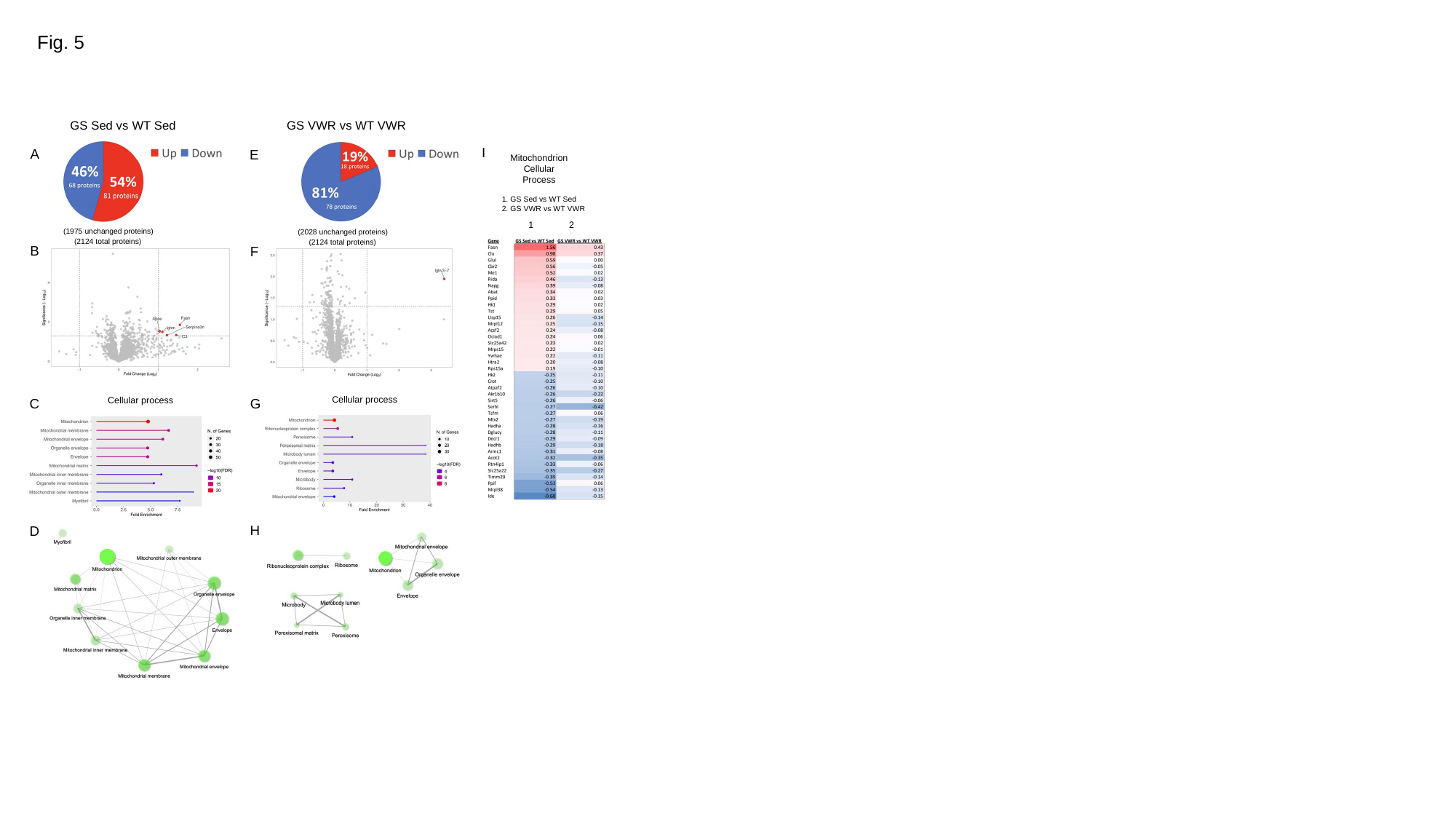

# Fig. 5
GS Sed vs WT Sed
GS VWR vs WT VWR
I
A
E
Mitochondrion
Cellular
Process
1. GS Sed vs WT Sed
2. GS VWR vs WT VWR
1 2
(1975 unchanged proteins)
(2028 unchanged proteins)
(2124 total proteins)
(2124 total proteins)
B
F
Cellular process
Cellular process
C
G
H
D

## Slide 6
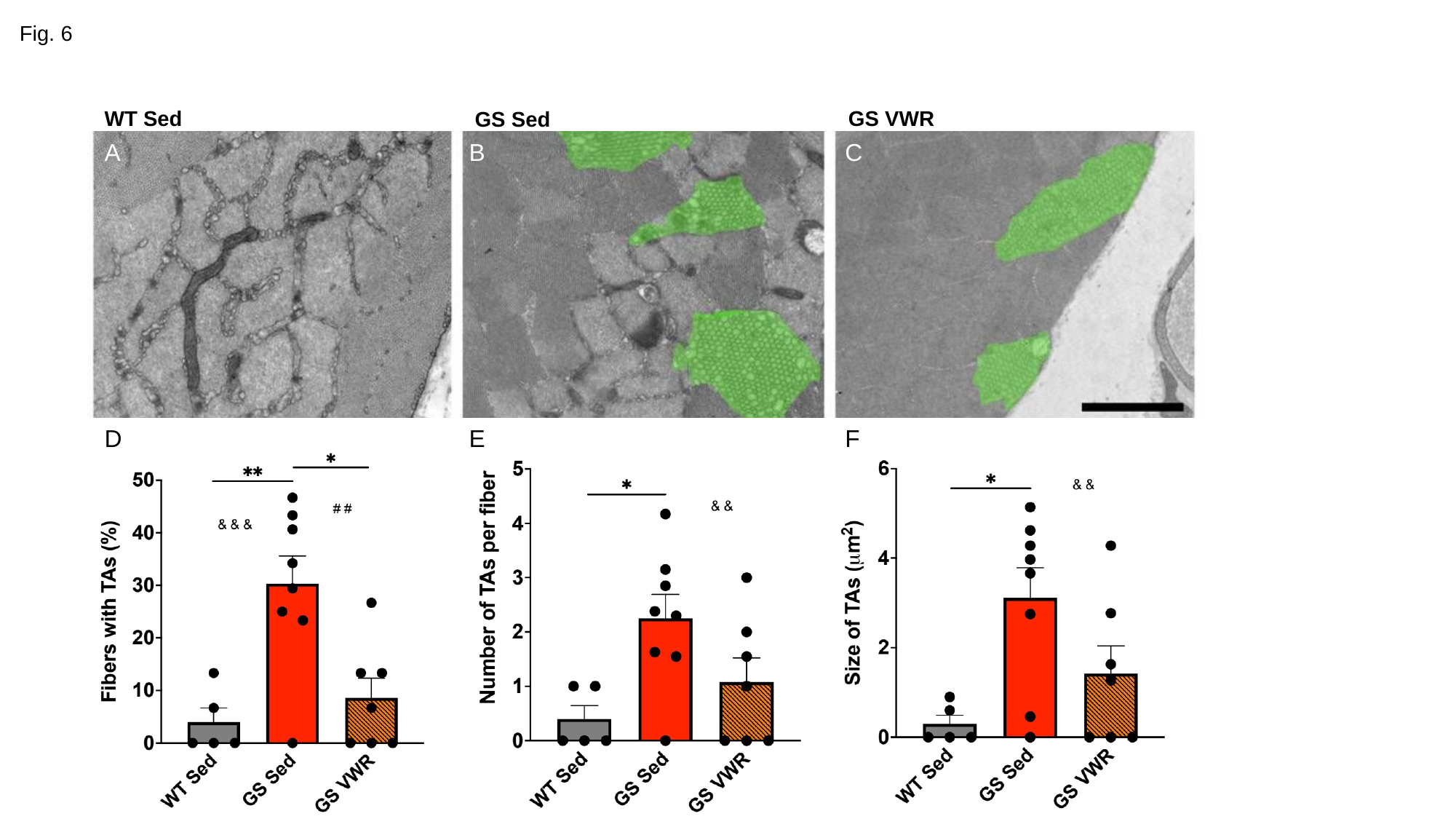

# Fig. 6
GS VWR
WT Sed
GS Sed
A
B
C
D
E
F

## Slide 7
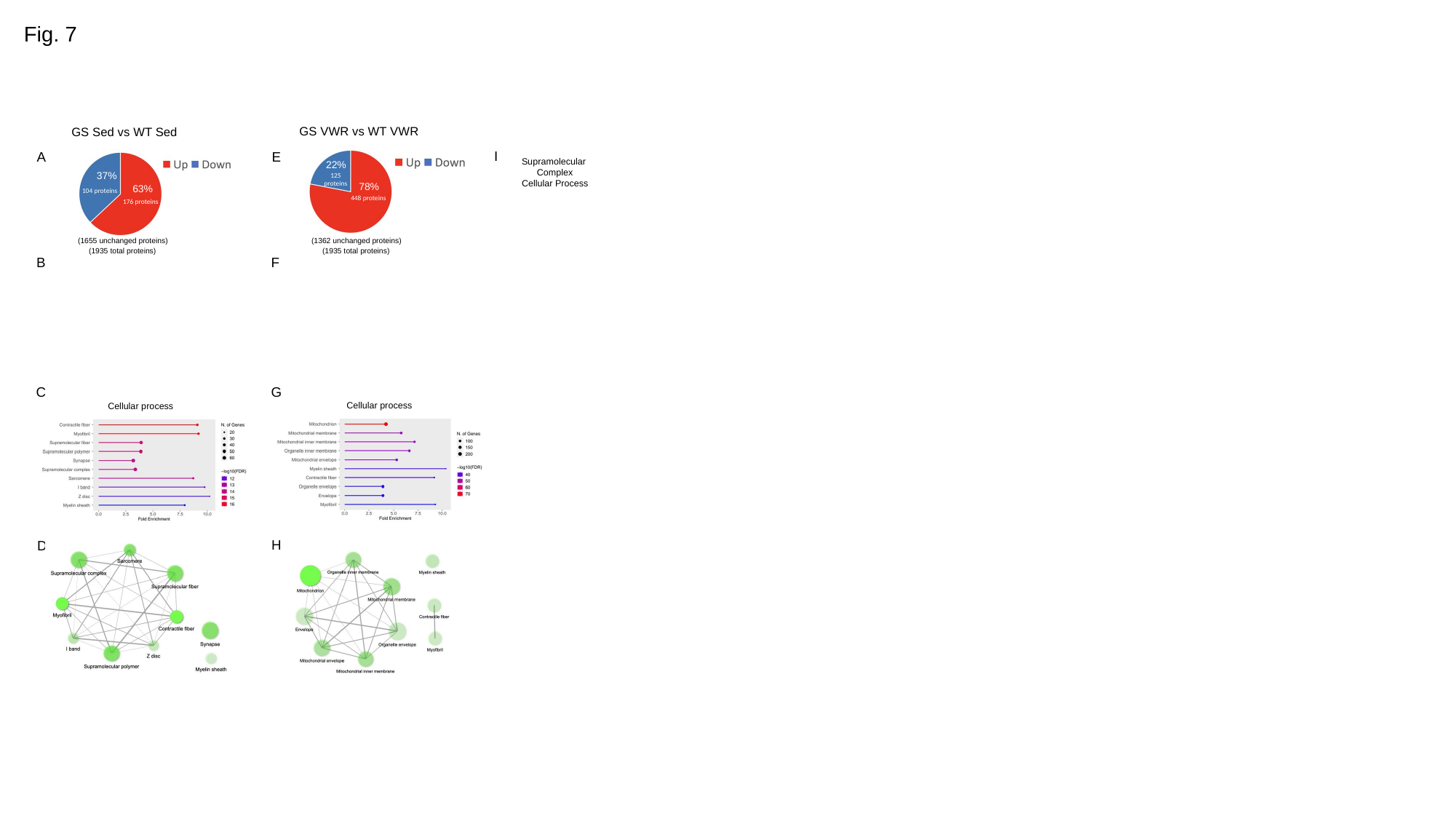

# Fig. 7
GS VWR vs WT VWR
GS Sed vs WT Sed
I
E
A
Supramolecular
Complex
Cellular Process
22%
37%
125 proteins
78%
63%
104 proteins
448 proteins
176 proteins
(1362 unchanged proteins)
(1655 unchanged proteins)
(1935 total proteins)
(1935 total proteins)
F
B
G
C
Cellular process
Cellular process
H
D

## Slide 8
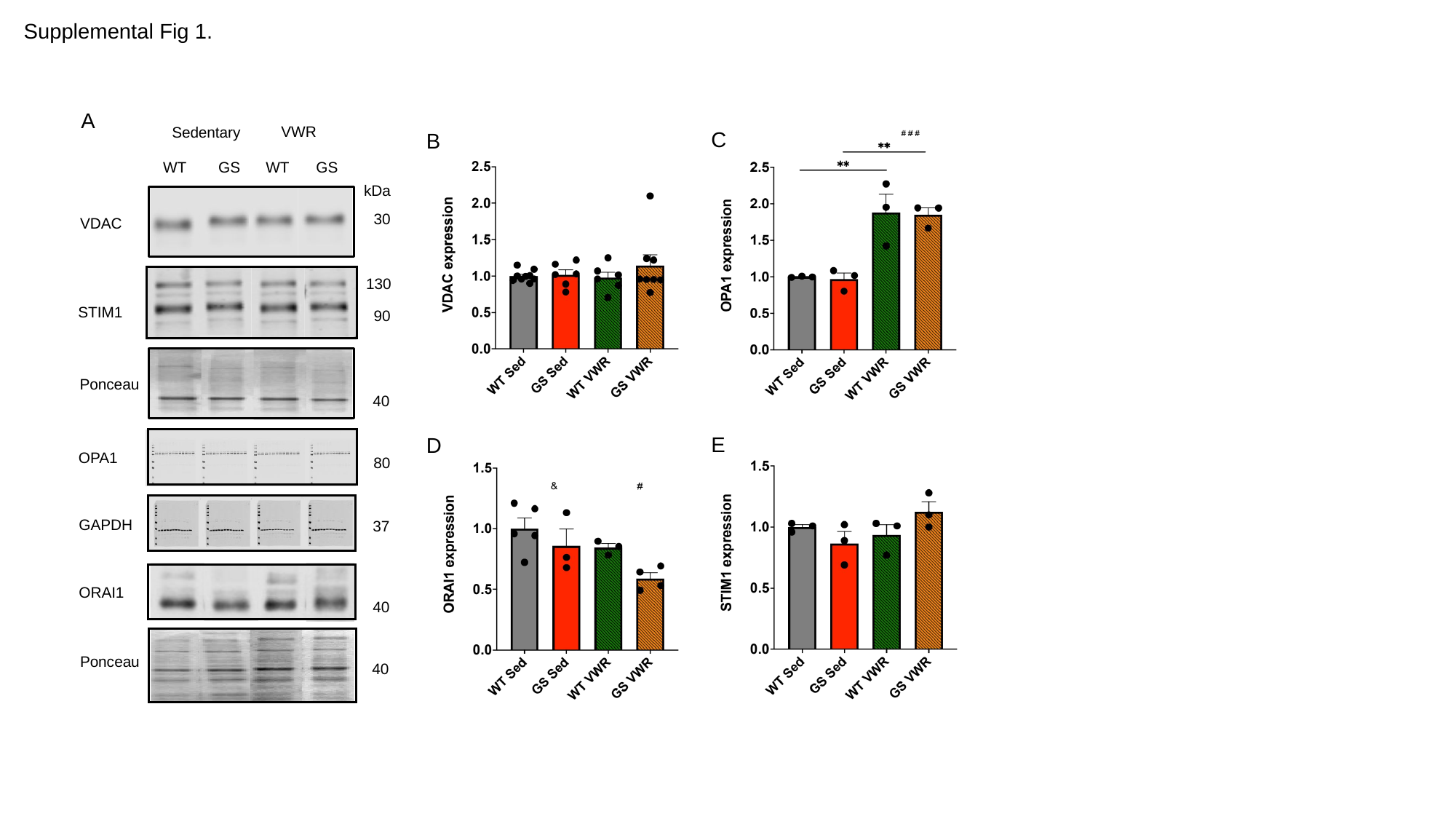

# Supplemental Fig 1.
A
VWR
Sedentary
C
B
GS
GS
WT
WT
kDa
30
VDAC
130
STIM1
90
Ponceau
40
E
D
OPA1
80
GAPDH
37
ORAI1
40
Ponceau
40

## Slide 9
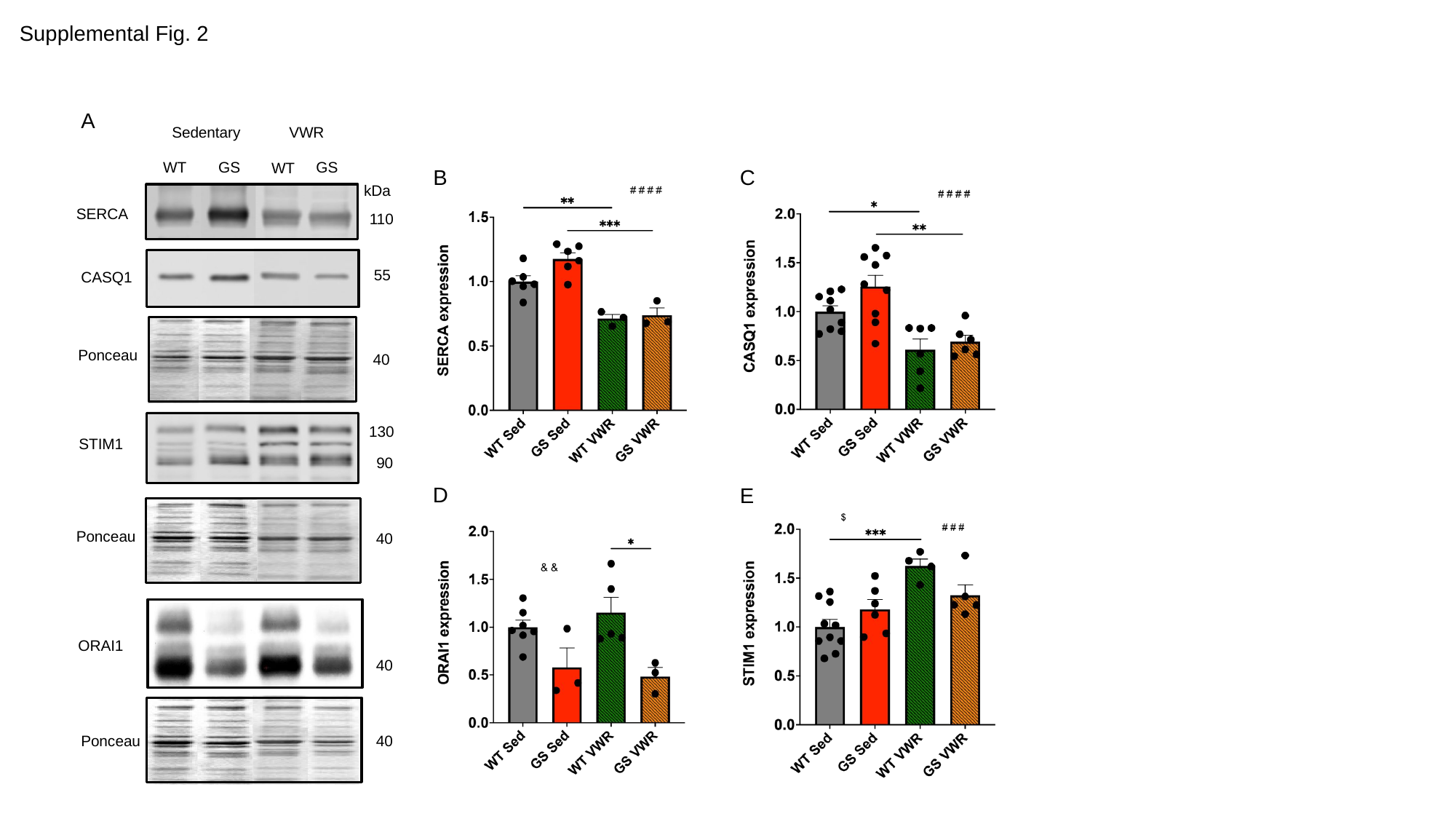

Supplemental Fig. 2
A
Sedentary
VWR
GS
GS
WT
WT
B
C
kDa
SERCA
110
55
CASQ1
Ponceau
40
130
STIM1
90
D
E
Ponceau
40
ORAI1
40
Ponceau
40

## Slide 10
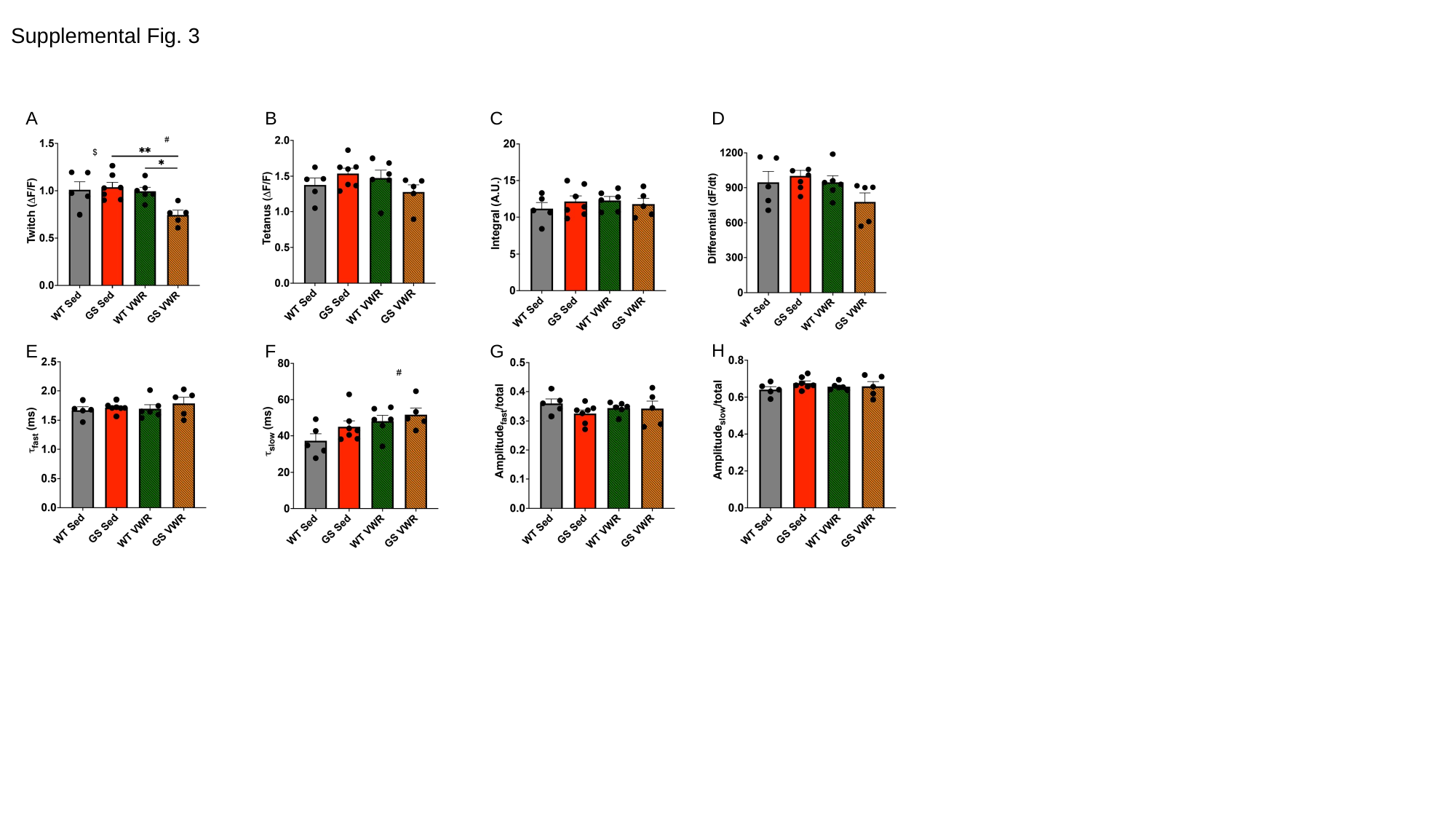

Supplemental Fig. 3
B
D
A
C
H
G
F
E

## Slide 11
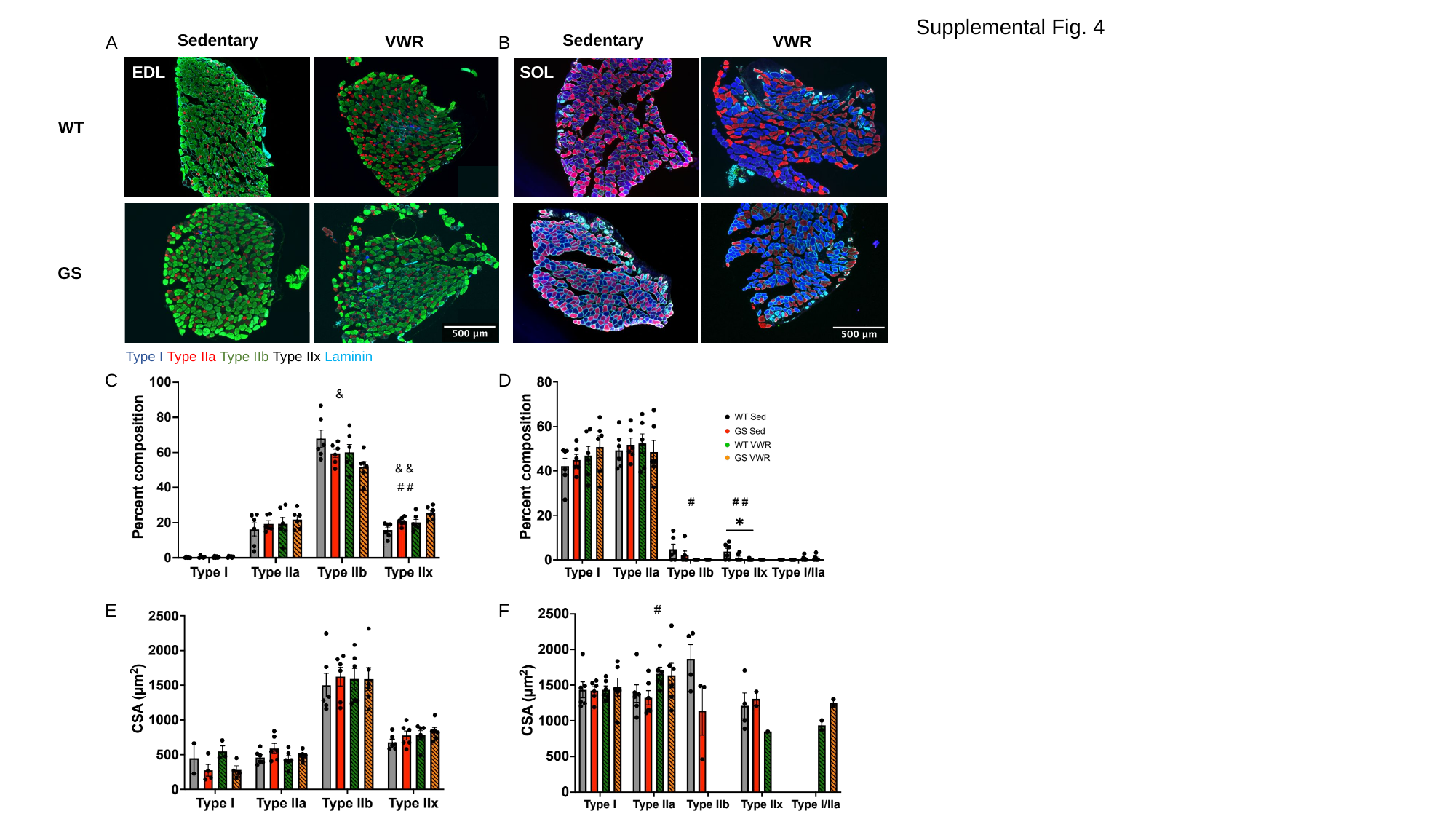

# Supplemental Fig. 4
Sedentary
Sedentary
VWR
VWR
B
A
EDL
SOL
WT
GS
Type I Type IIa Type IIb Type IIx Laminin
C
D
F
E

## Slide 12
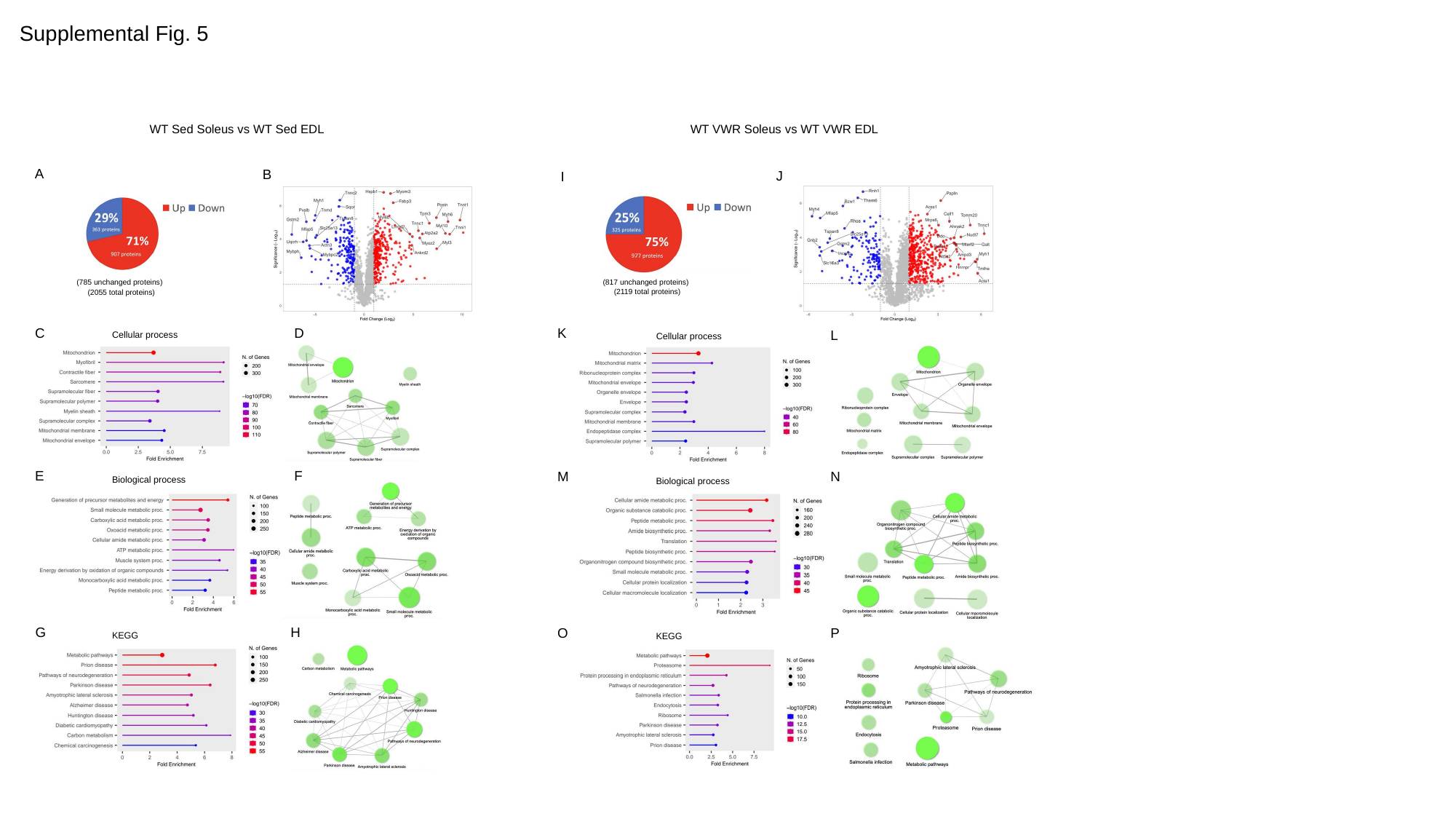

Supplemental Fig. 5
WT Sed Soleus vs WT Sed EDL
WT VWR Soleus vs WT VWR EDL
A
B
J
I
(817 unchanged proteins)
(785 unchanged proteins)
(2119 total proteins)
(2055 total proteins)
C
D
K
L
Cellular process
Cellular process
E
F
M
N
Biological process
Biological process
G
H
P
O
KEGG
KEGG

## Slide 13
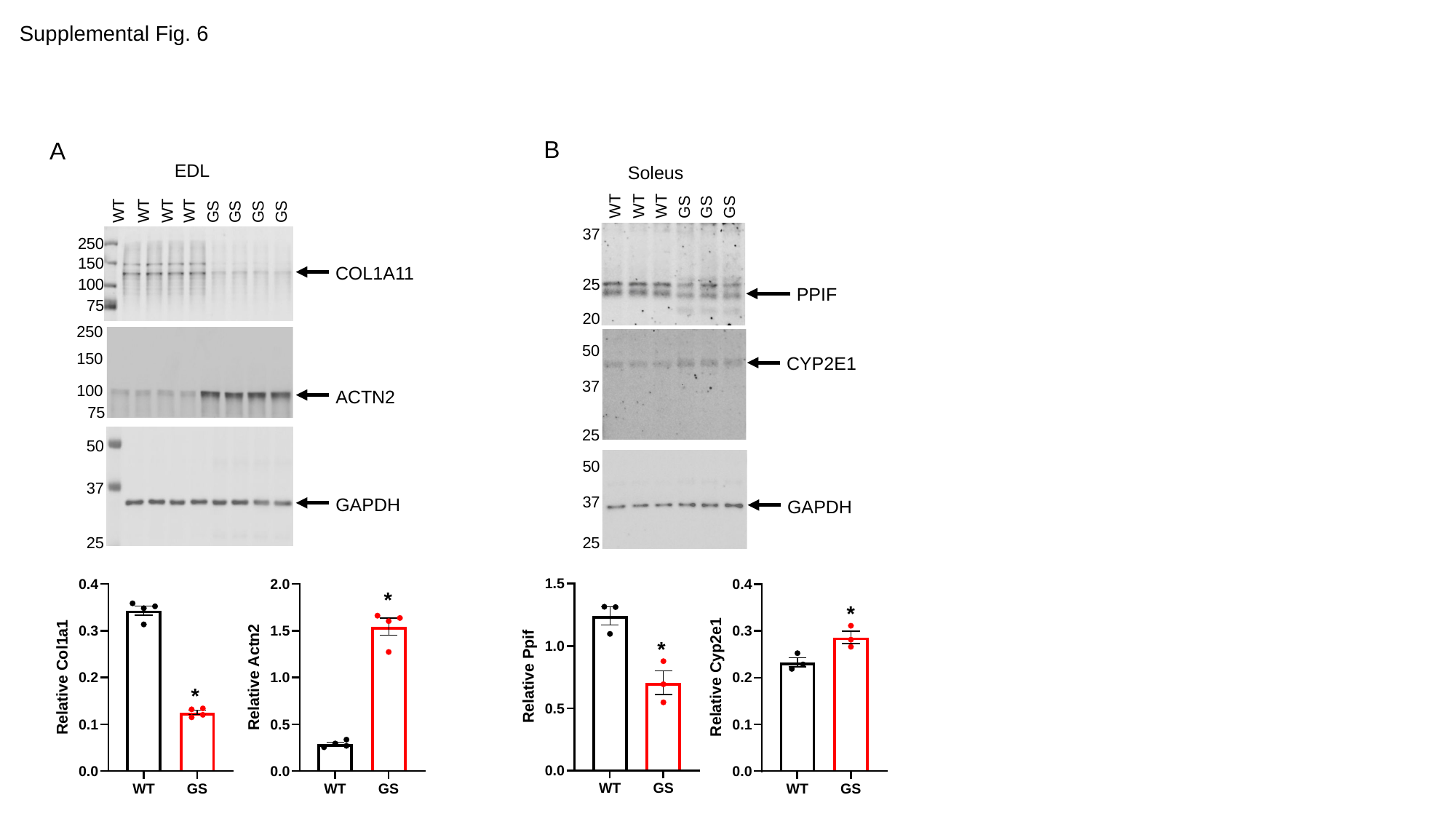

Supplemental Fig. 6
B
A
EDL
Soleus
WT
WT
WT
GS
GS
GS
37
25
20
PPIF
50
37
25
CYP2E1
50
37
25
GAPDH
WT
WT
WT
WT
GS
GS
GS
GS
250
150
100
75
COL1A11
250
150
100
75
ACTN2
50
37
25
GAPDH
*
*
*
*

## Slide 14
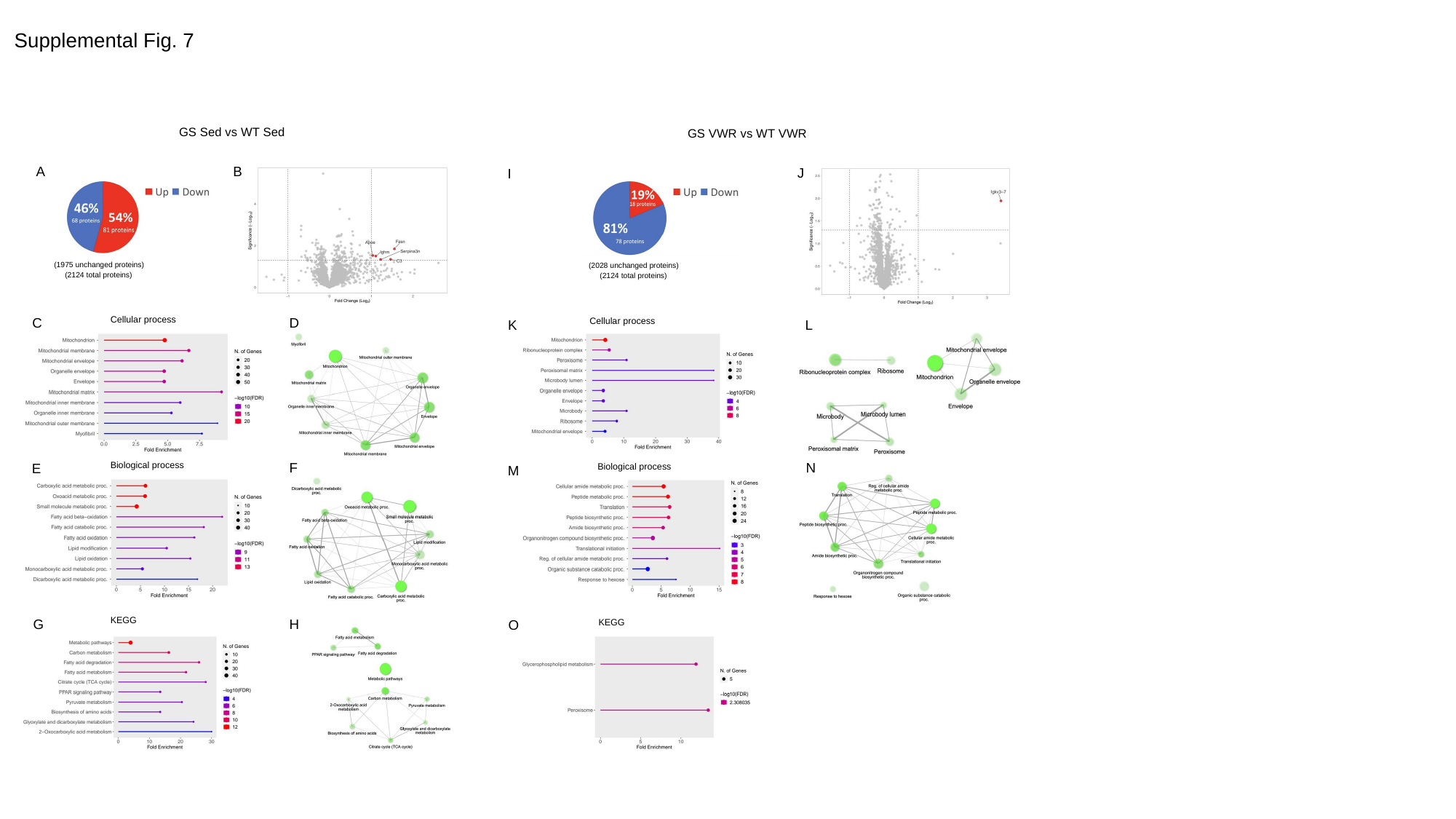

# Supplemental Fig. 7
GS Sed vs WT Sed
GS VWR vs WT VWR
B
A
J
I
(1975 unchanged proteins)
(2028 unchanged proteins)
(2124 total proteins)
(2124 total proteins)
Cellular process
C
D
Cellular process
K
L
Biological process
F
N
E
Biological process
M
KEGG
G
H
KEGG
O

## Slide 15
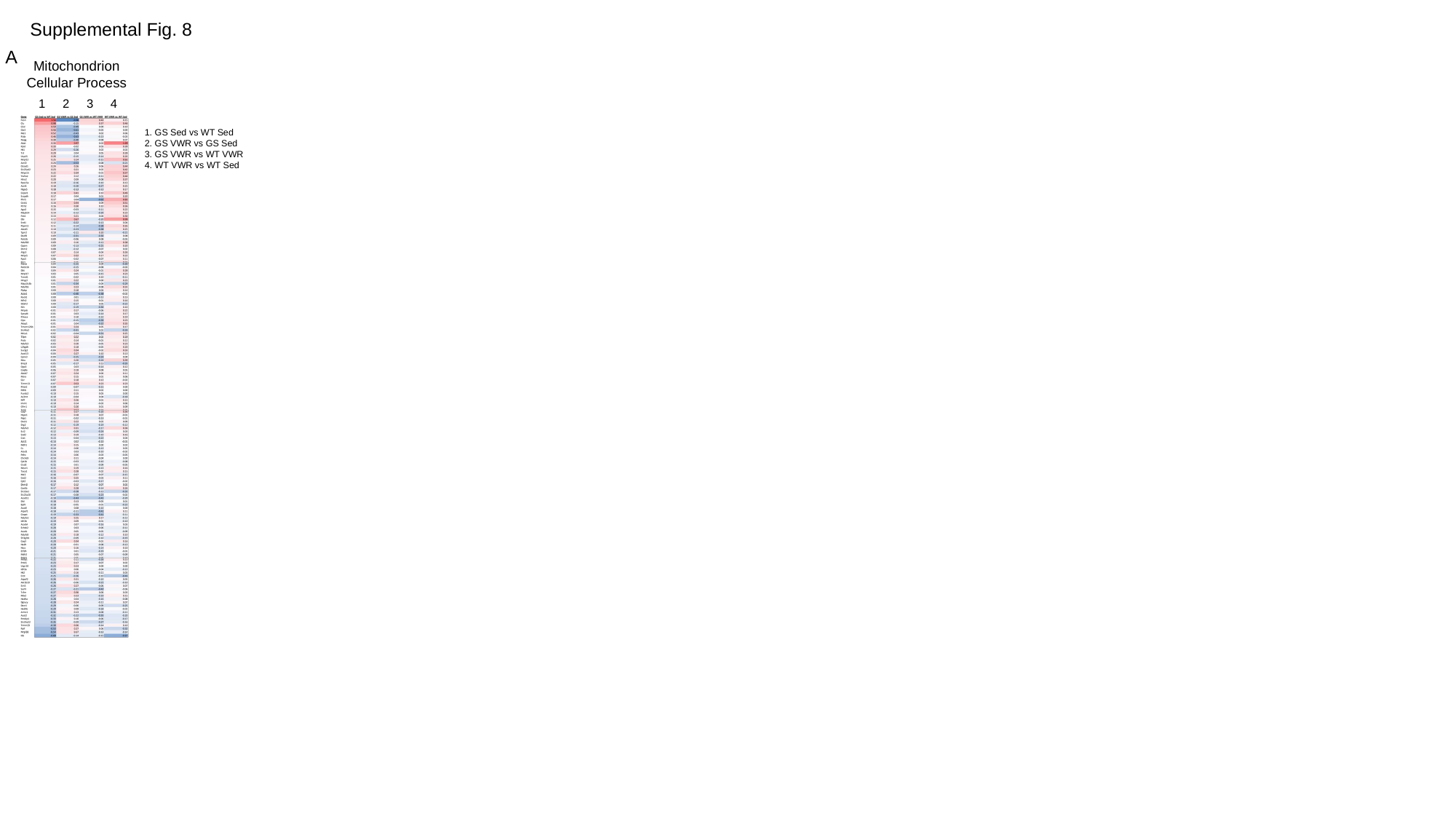

# Supplemental Fig. 8
A
Mitochondrion
Cellular Process
1 2 3 4
1. GS Sed vs WT Sed
2. GS VWR vs GS Sed
3. GS VWR vs WT VWR
4. WT VWR vs WT Sed

## Slide 16
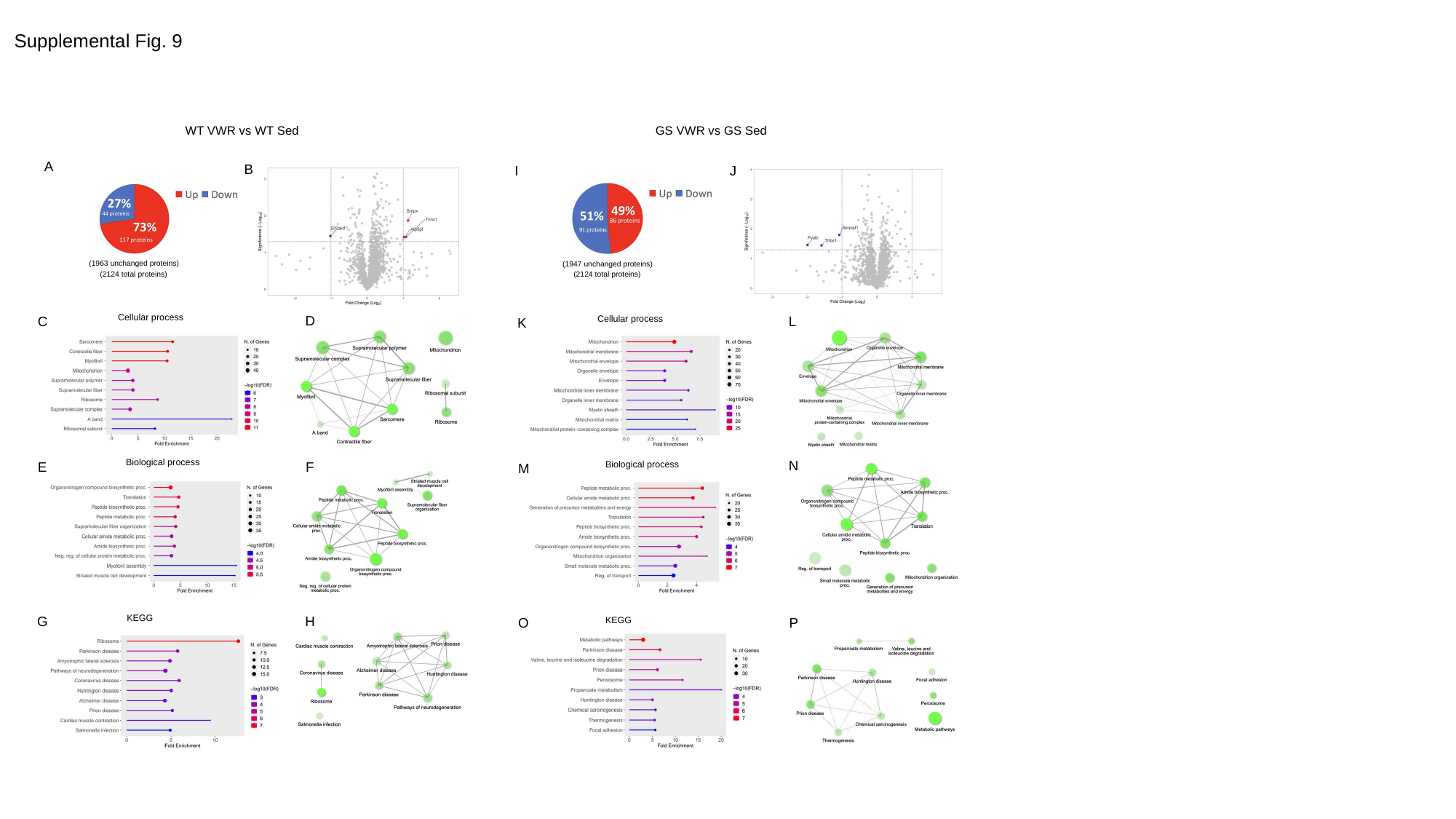

# Supplemental Fig. 9
WT VWR vs WT Sed
GS VWR vs GS Sed
A
B
J
I
(1963 unchanged proteins)
(1947 unchanged proteins)
(2124 total proteins)
(2124 total proteins)
Cellular process
D
C
L
Cellular process
K
N
Biological process
E
F
Biological process
M
H
KEGG
G
O
P
KEGG

## Slide 17
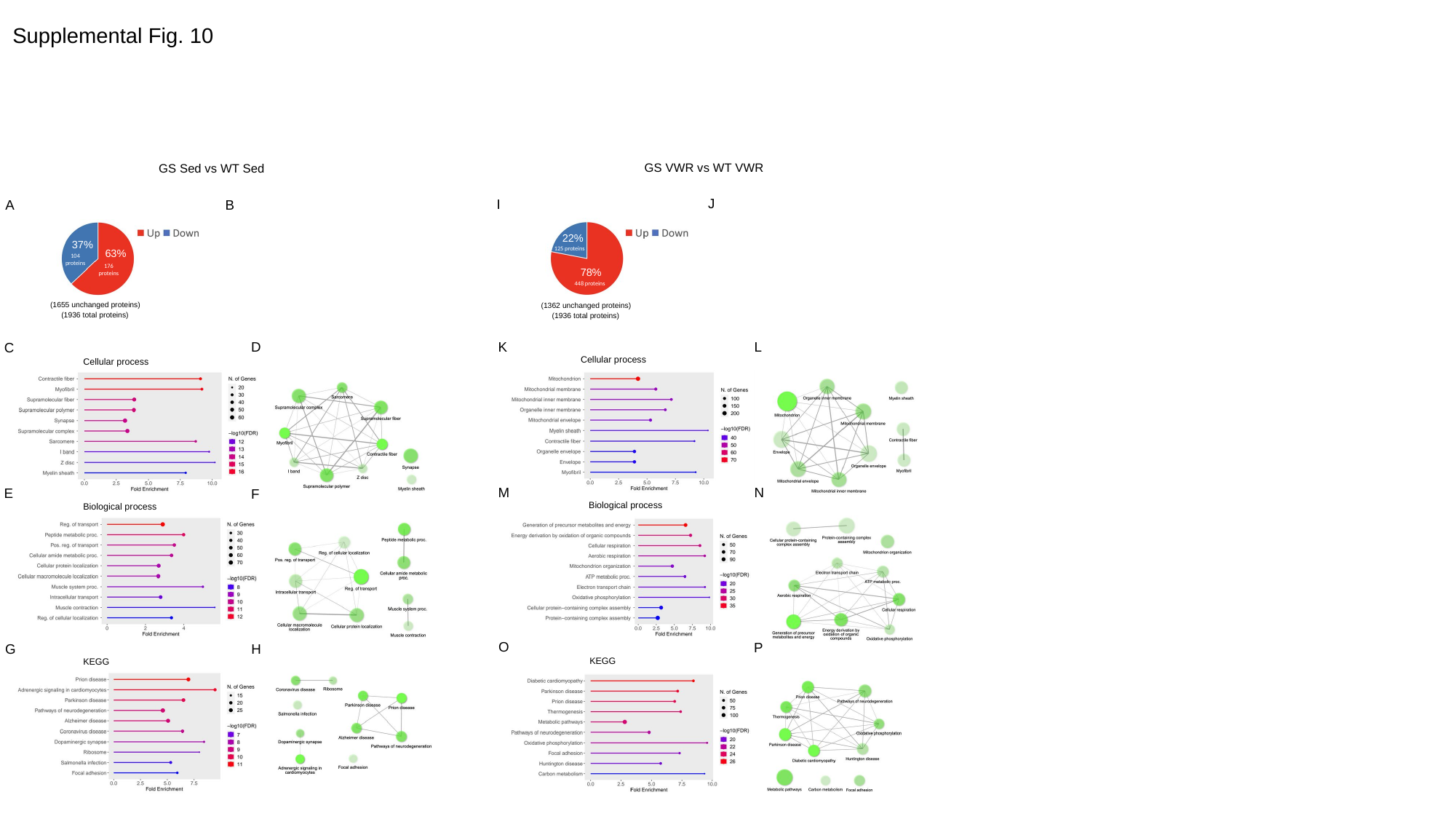

# Supplemental Fig. 10
GS VWR vs WT VWR
GS Sed vs WT Sed
J
I
B
A
22%
37%
125 proteins
63%
104 proteins
176 proteins
78%
448 proteins
(1655 unchanged proteins)
(1362 unchanged proteins)
(1936 total proteins)
(1936 total proteins)
K
D
L
C
Cellular process
Cellular process
M
N
E
F
Biological process
Biological process
O
P
G
H
KEGG
KEGG

## Slide 18
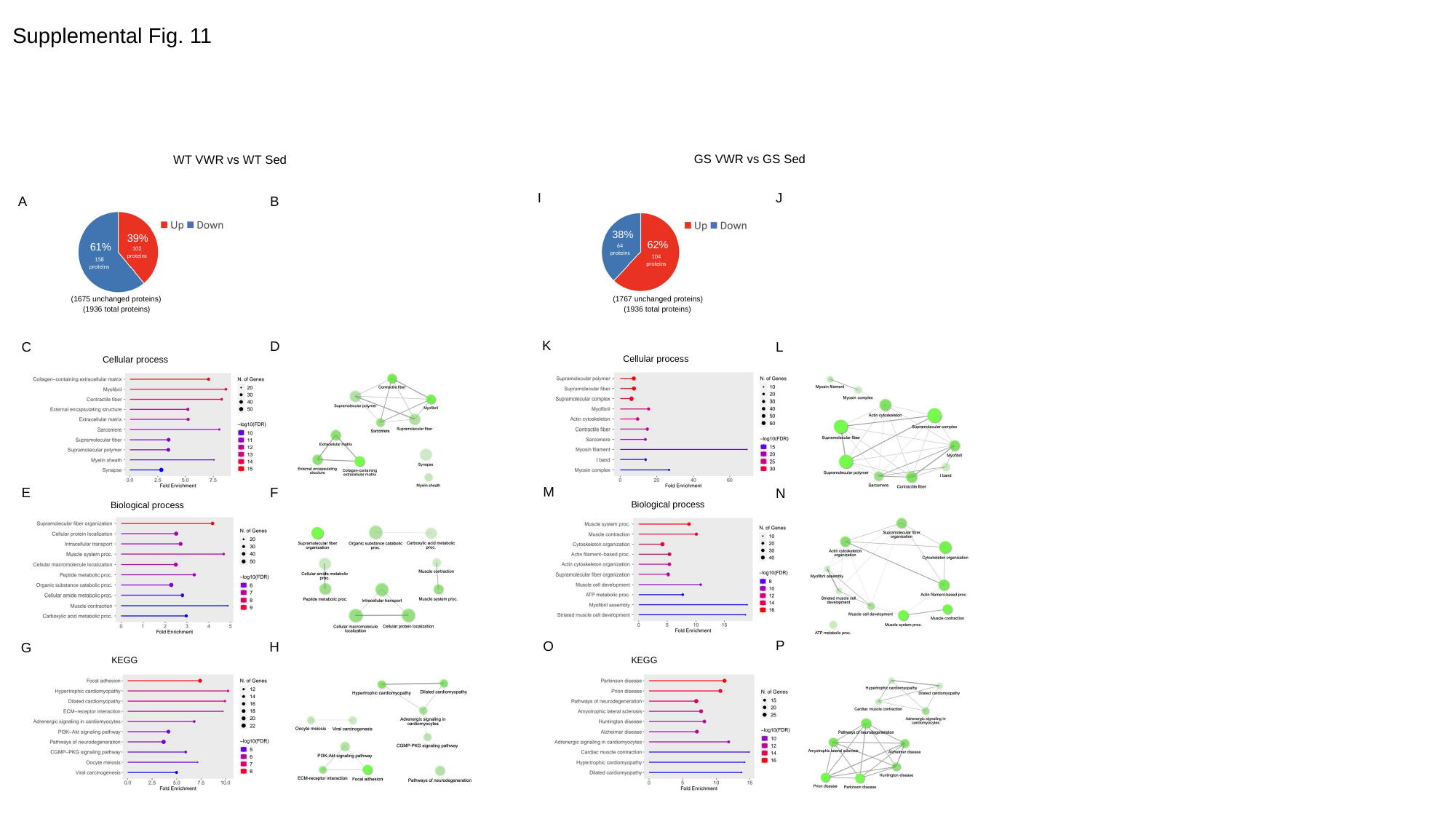

# Supplemental Fig. 11
GS VWR vs GS Sed
WT VWR vs WT Sed
J
I
B
A
38%
39%
62%
61%
64 proteins
102 proteins
104 proteins
158 proteins
(1767 unchanged proteins)
(1675 unchanged proteins)
(1936 total proteins)
(1936 total proteins)
K
D
C
L
Cellular process
Cellular process
M
F
E
N
Biological process
Biological process
P
O
H
G
KEGG
KEGG

## Slide 19
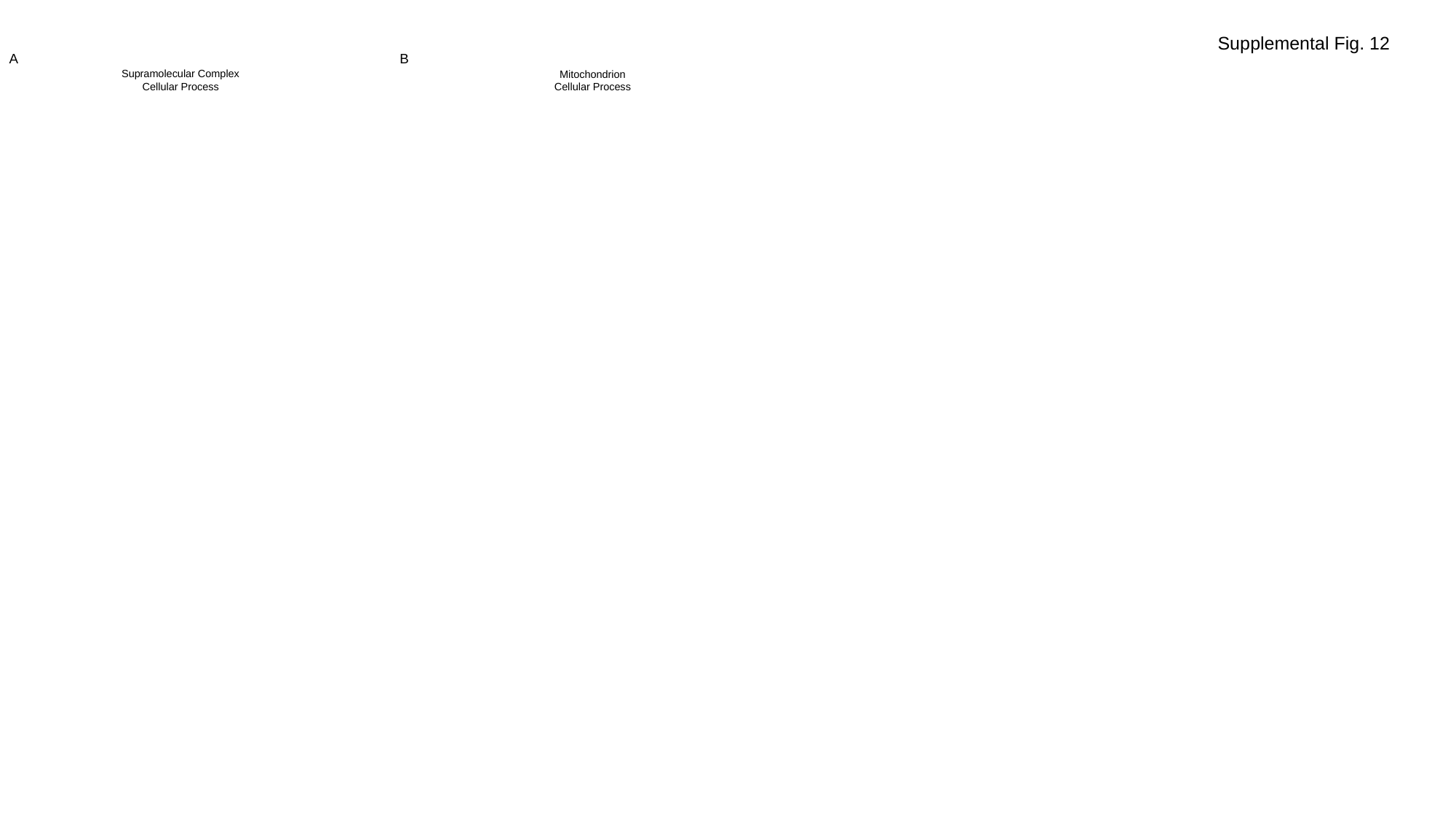

# Supplemental Fig. 12
A
B
Supramolecular Complex
Cellular Process
Mitochondrion
Cellular Process

## Slide 20
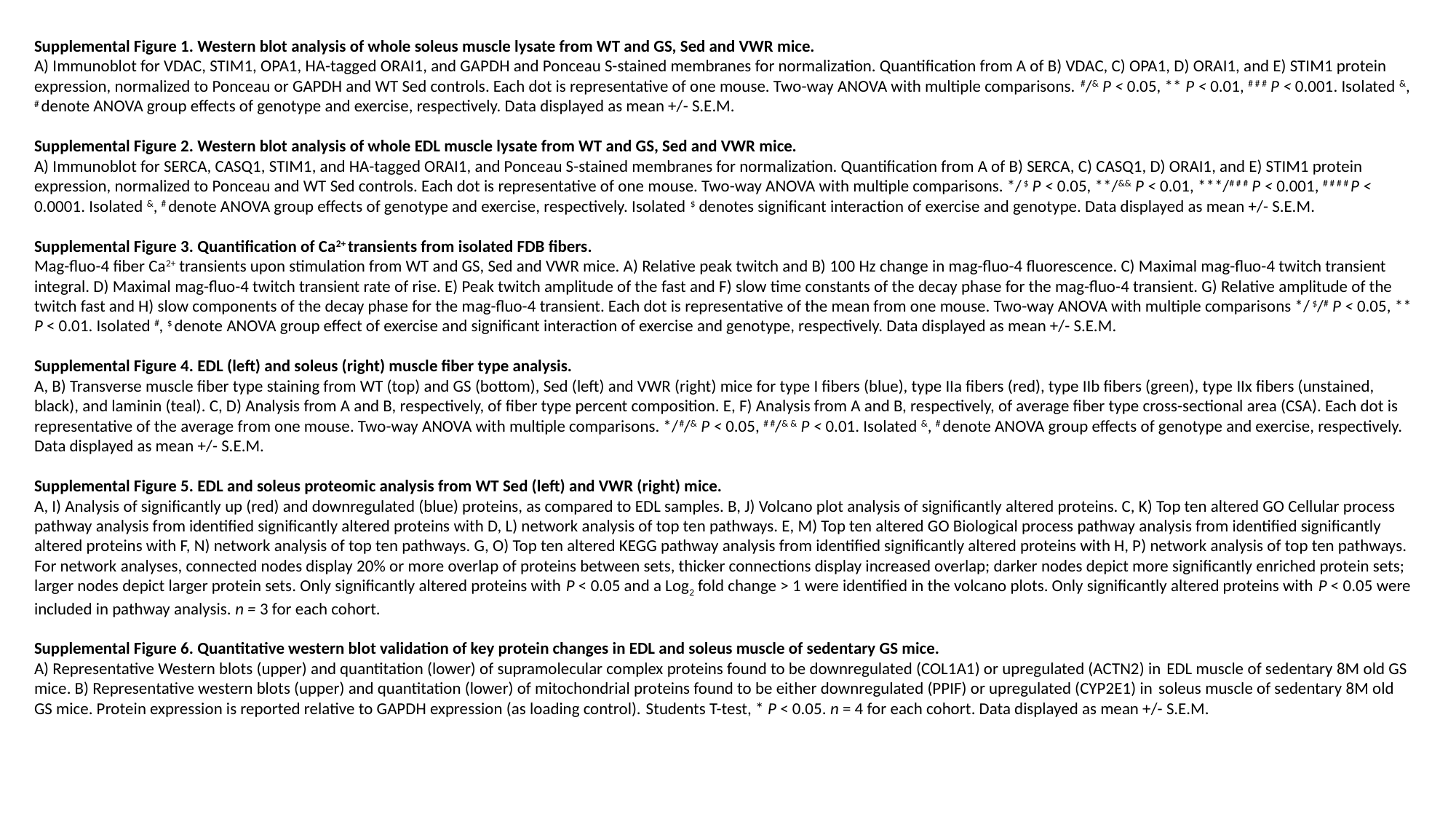

Supplemental Figure 1. Western blot analysis of whole soleus muscle lysate from WT and GS, Sed and VWR mice.
A) Immunoblot for VDAC, STIM1, OPA1, HA-tagged ORAI1, and GAPDH and Ponceau S-stained membranes for normalization. Quantification from A of B) VDAC, C) OPA1, D) ORAI1, and E) STIM1 protein expression, normalized to Ponceau or GAPDH and WT Sed controls. Each dot is representative of one mouse. Two-way ANOVA with multiple comparisons. #/& P < 0.05, ** P < 0.01, # # # P < 0.001. Isolated &, # denote ANOVA group effects of genotype and exercise, respectively. Data displayed as mean +/- S.E.M.
Supplemental Figure 2. Western blot analysis of whole EDL muscle lysate from WT and GS, Sed and VWR mice.
A) Immunoblot for SERCA, CASQ1, STIM1, and HA-tagged ORAI1, and Ponceau S-stained membranes for normalization. Quantification from A of B) SERCA, C) CASQ1, D) ORAI1, and E) STIM1 protein expression, normalized to Ponceau and WT Sed controls. Each dot is representative of one mouse. Two-way ANOVA with multiple comparisons. */$ P < 0.05, **/&& P < 0.01, ***/# # # P < 0.001, # # # # P < 0.0001. Isolated &, # denote ANOVA group effects of genotype and exercise, respectively. Isolated $ denotes significant interaction of exercise and genotype. Data displayed as mean +/- S.E.M.
Supplemental Figure 3. Quantification of Ca2+ transients from isolated FDB fibers.
Mag-fluo-4 fiber Ca2+ transients upon stimulation from WT and GS, Sed and VWR mice. A) Relative peak twitch and B) 100 Hz change in mag-fluo-4 fluorescence. C) Maximal mag-fluo-4 twitch transient integral. D) Maximal mag-fluo-4 twitch transient rate of rise. E) Peak twitch amplitude of the fast and F) slow time constants of the decay phase for the mag-fluo-4 transient. G) Relative amplitude of the twitch fast and H) slow components of the decay phase for the mag-fluo-4 transient. Each dot is representative of the mean from one mouse. Two-way ANOVA with multiple comparisons */$/# P < 0.05, ** P < 0.01. Isolated #, $ denote ANOVA group effect of exercise and significant interaction of exercise and genotype, respectively. Data displayed as mean +/- S.E.M.
Supplemental Figure 4. EDL (left) and soleus (right) muscle fiber type analysis.
A, B) Transverse muscle fiber type staining from WT (top) and GS (bottom), Sed (left) and VWR (right) mice for type I fibers (blue), type IIa fibers (red), type IIb fibers (green), type IIx fibers (unstained, black), and laminin (teal). C, D) Analysis from A and B, respectively, of fiber type percent composition. E, F) Analysis from A and B, respectively, of average fiber type cross-sectional area (CSA). Each dot is representative of the average from one mouse. Two-way ANOVA with multiple comparisons. */#/& P < 0.05, # #/& & P < 0.01. Isolated &, # denote ANOVA group effects of genotype and exercise, respectively. Data displayed as mean +/- S.E.M.
Supplemental Figure 5. EDL and soleus proteomic analysis from WT Sed (left) and VWR (right) mice.
A, I) Analysis of significantly up (red) and downregulated (blue) proteins, as compared to EDL samples. B, J) Volcano plot analysis of significantly altered proteins. C, K) Top ten altered GO Cellular process pathway analysis from identified significantly altered proteins with D, L) network analysis of top ten pathways. E, M) Top ten altered GO Biological process pathway analysis from identified significantly altered proteins with F, N) network analysis of top ten pathways. G, O) Top ten altered KEGG pathway analysis from identified significantly altered proteins with H, P) network analysis of top ten pathways. For network analyses, connected nodes display 20% or more overlap of proteins between sets, thicker connections display increased overlap; darker nodes depict more significantly enriched protein sets; larger nodes depict larger protein sets. Only significantly altered proteins with P < 0.05 and a Log2 fold change > 1 were identified in the volcano plots. Only significantly altered proteins with P < 0.05 were included in pathway analysis. n = 3 for each cohort.
Supplemental Figure 6. Quantitative western blot validation of key protein changes in EDL and soleus muscle of sedentary GS mice.
A) Representative Western blots (upper) and quantitation (lower) of supramolecular complex proteins found to be downregulated (COL1A1) or upregulated (ACTN2) in EDL muscle of sedentary 8M old GS mice. B) Representative western blots (upper) and quantitation (lower) of mitochondrial proteins found to be either downregulated (PPIF) or upregulated (CYP2E1) in soleus muscle of sedentary 8M old GS mice. Protein expression is reported relative to GAPDH expression (as loading control). Students T-test, * P < 0.05. n = 4 for each cohort. Data displayed as mean +/- S.E.M.

## Slide 21
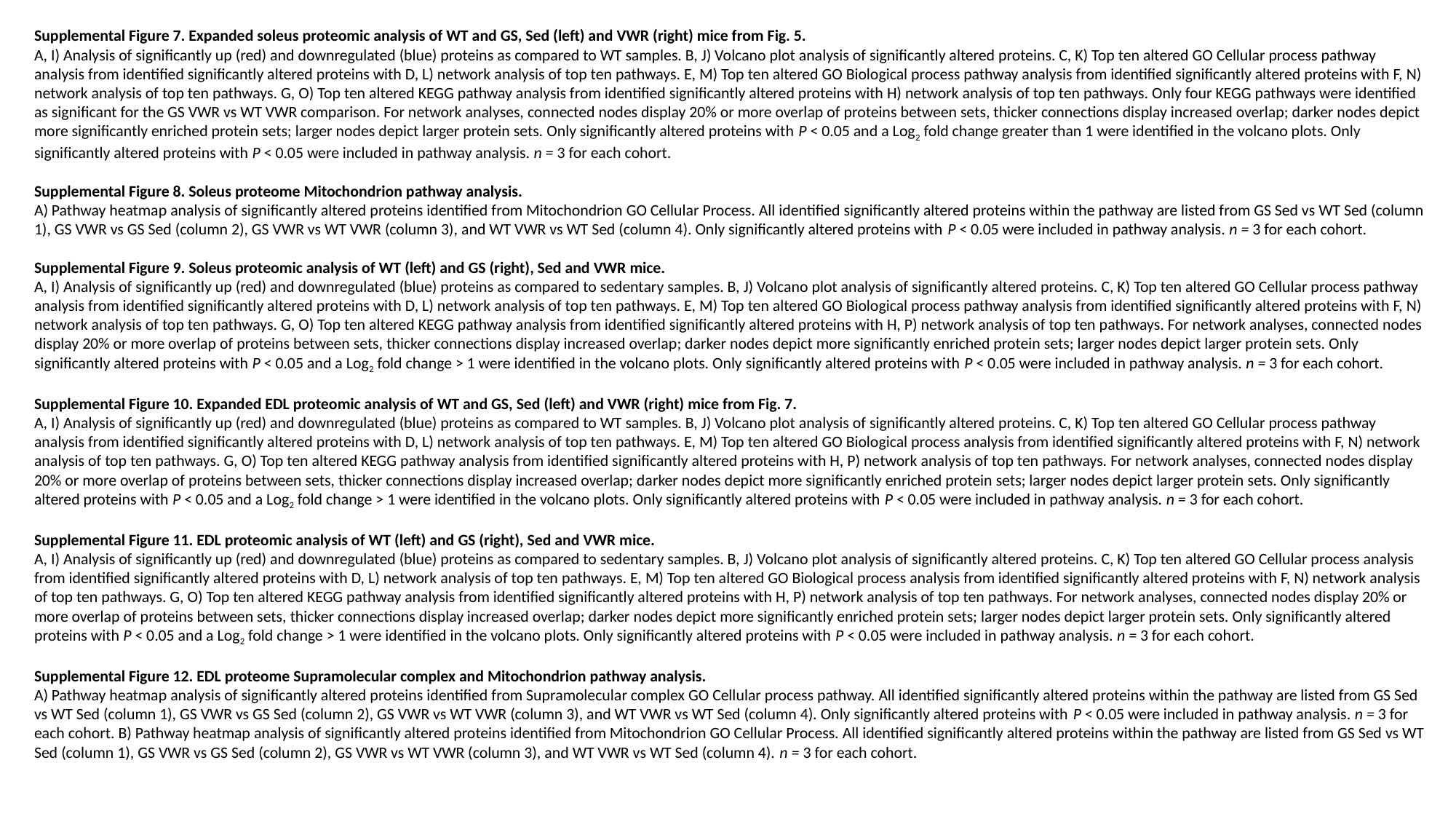

Supplemental Figure 7. Expanded soleus proteomic analysis of WT and GS, Sed (left) and VWR (right) mice from Fig. 5.
A, I) Analysis of significantly up (red) and downregulated (blue) proteins as compared to WT samples. B, J) Volcano plot analysis of significantly altered proteins. C, K) Top ten altered GO Cellular process pathway analysis from identified significantly altered proteins with D, L) network analysis of top ten pathways. E, M) Top ten altered GO Biological process pathway analysis from identified significantly altered proteins with F, N) network analysis of top ten pathways. G, O) Top ten altered KEGG pathway analysis from identified significantly altered proteins with H) network analysis of top ten pathways. Only four KEGG pathways were identified as significant for the GS VWR vs WT VWR comparison. For network analyses, connected nodes display 20% or more overlap of proteins between sets, thicker connections display increased overlap; darker nodes depict more significantly enriched protein sets; larger nodes depict larger protein sets. Only significantly altered proteins with P < 0.05 and a Log2 fold change greater than 1 were identified in the volcano plots. Only significantly altered proteins with P < 0.05 were included in pathway analysis. n = 3 for each cohort.
Supplemental Figure 8. Soleus proteome Mitochondrion pathway analysis.
A) Pathway heatmap analysis of significantly altered proteins identified from Mitochondrion GO Cellular Process. All identified significantly altered proteins within the pathway are listed from GS Sed vs WT Sed (column 1), GS VWR vs GS Sed (column 2), GS VWR vs WT VWR (column 3), and WT VWR vs WT Sed (column 4). Only significantly altered proteins with P < 0.05 were included in pathway analysis. n = 3 for each cohort.
Supplemental Figure 9. Soleus proteomic analysis of WT (left) and GS (right), Sed and VWR mice.
A, I) Analysis of significantly up (red) and downregulated (blue) proteins as compared to sedentary samples. B, J) Volcano plot analysis of significantly altered proteins. C, K) Top ten altered GO Cellular process pathway analysis from identified significantly altered proteins with D, L) network analysis of top ten pathways. E, M) Top ten altered GO Biological process pathway analysis from identified significantly altered proteins with F, N) network analysis of top ten pathways. G, O) Top ten altered KEGG pathway analysis from identified significantly altered proteins with H, P) network analysis of top ten pathways. For network analyses, connected nodes display 20% or more overlap of proteins between sets, thicker connections display increased overlap; darker nodes depict more significantly enriched protein sets; larger nodes depict larger protein sets. Only significantly altered proteins with P < 0.05 and a Log2 fold change > 1 were identified in the volcano plots. Only significantly altered proteins with P < 0.05 were included in pathway analysis. n = 3 for each cohort.
Supplemental Figure 10. Expanded EDL proteomic analysis of WT and GS, Sed (left) and VWR (right) mice from Fig. 7.
A, I) Analysis of significantly up (red) and downregulated (blue) proteins as compared to WT samples. B, J) Volcano plot analysis of significantly altered proteins. C, K) Top ten altered GO Cellular process pathway analysis from identified significantly altered proteins with D, L) network analysis of top ten pathways. E, M) Top ten altered GO Biological process analysis from identified significantly altered proteins with F, N) network analysis of top ten pathways. G, O) Top ten altered KEGG pathway analysis from identified significantly altered proteins with H, P) network analysis of top ten pathways. For network analyses, connected nodes display 20% or more overlap of proteins between sets, thicker connections display increased overlap; darker nodes depict more significantly enriched protein sets; larger nodes depict larger protein sets. Only significantly altered proteins with P < 0.05 and a Log2 fold change > 1 were identified in the volcano plots. Only significantly altered proteins with P < 0.05 were included in pathway analysis. n = 3 for each cohort.
Supplemental Figure 11. EDL proteomic analysis of WT (left) and GS (right), Sed and VWR mice.
A, I) Analysis of significantly up (red) and downregulated (blue) proteins as compared to sedentary samples. B, J) Volcano plot analysis of significantly altered proteins. C, K) Top ten altered GO Cellular process analysis from identified significantly altered proteins with D, L) network analysis of top ten pathways. E, M) Top ten altered GO Biological process analysis from identified significantly altered proteins with F, N) network analysis of top ten pathways. G, O) Top ten altered KEGG pathway analysis from identified significantly altered proteins with H, P) network analysis of top ten pathways. For network analyses, connected nodes display 20% or more overlap of proteins between sets, thicker connections display increased overlap; darker nodes depict more significantly enriched protein sets; larger nodes depict larger protein sets. Only significantly altered proteins with P < 0.05 and a Log2 fold change > 1 were identified in the volcano plots. Only significantly altered proteins with P < 0.05 were included in pathway analysis. n = 3 for each cohort.
Supplemental Figure 12. EDL proteome Supramolecular complex and Mitochondrion pathway analysis.
A) Pathway heatmap analysis of significantly altered proteins identified from Supramolecular complex GO Cellular process pathway. All identified significantly altered proteins within the pathway are listed from GS Sed vs WT Sed (column 1), GS VWR vs GS Sed (column 2), GS VWR vs WT VWR (column 3), and WT VWR vs WT Sed (column 4). Only significantly altered proteins with P < 0.05 were included in pathway analysis. n = 3 for each cohort. B) Pathway heatmap analysis of significantly altered proteins identified from Mitochondrion GO Cellular Process. All identified significantly altered proteins within the pathway are listed from GS Sed vs WT Sed (column 1), GS VWR vs GS Sed (column 2), GS VWR vs WT VWR (column 3), and WT VWR vs WT Sed (column 4). n = 3 for each cohort.

## Slide 22
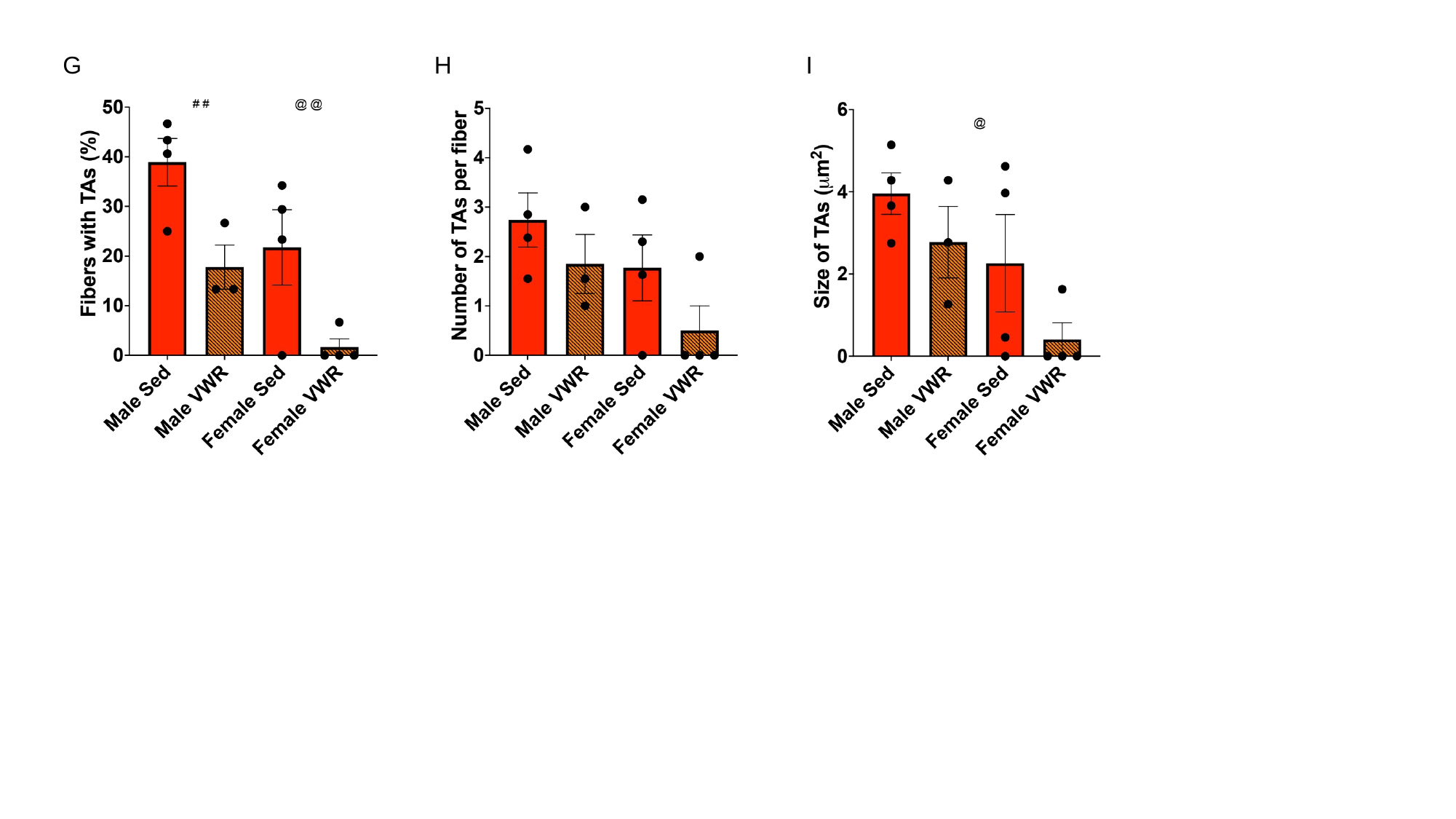

G
H
I
