# Supplementary figures and images for: Voluntary Wheel Running Mitigates Disease in an Orai1 Gain-of-Function Mouse Model of Tubular Aggregate Myopathy
Source: Cells. 2025 Sep 4;14(17):1383. doi: 10.3390/cells14171383 (PMC12427812; doi:10.3390/cells14171383)

Fig. 1

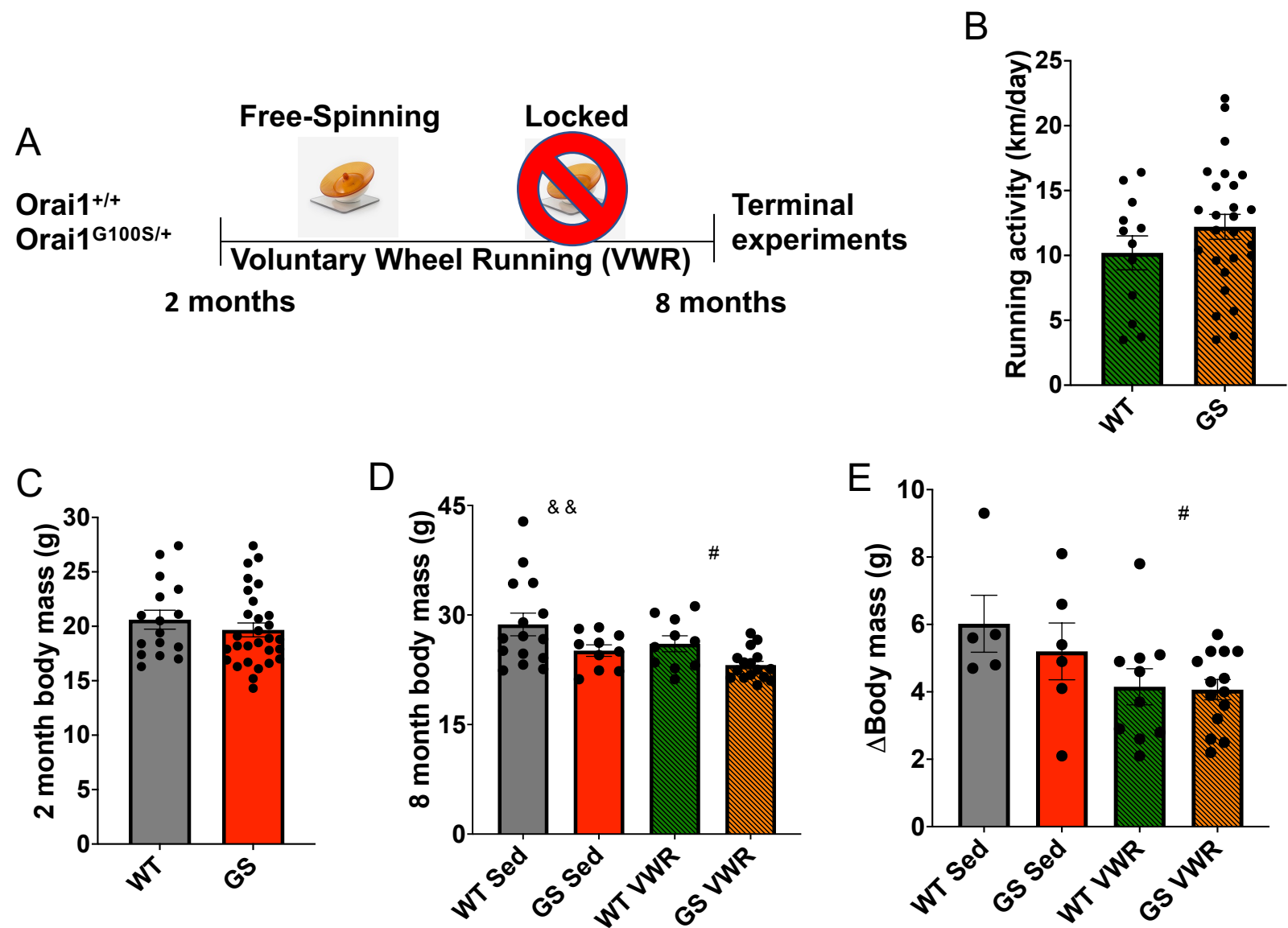

Supplement: Supplementary file 1 [file cells-14-01383-s001.zip › GS VWR Figures 20250821 Fig1.pdf]

Fig. 2

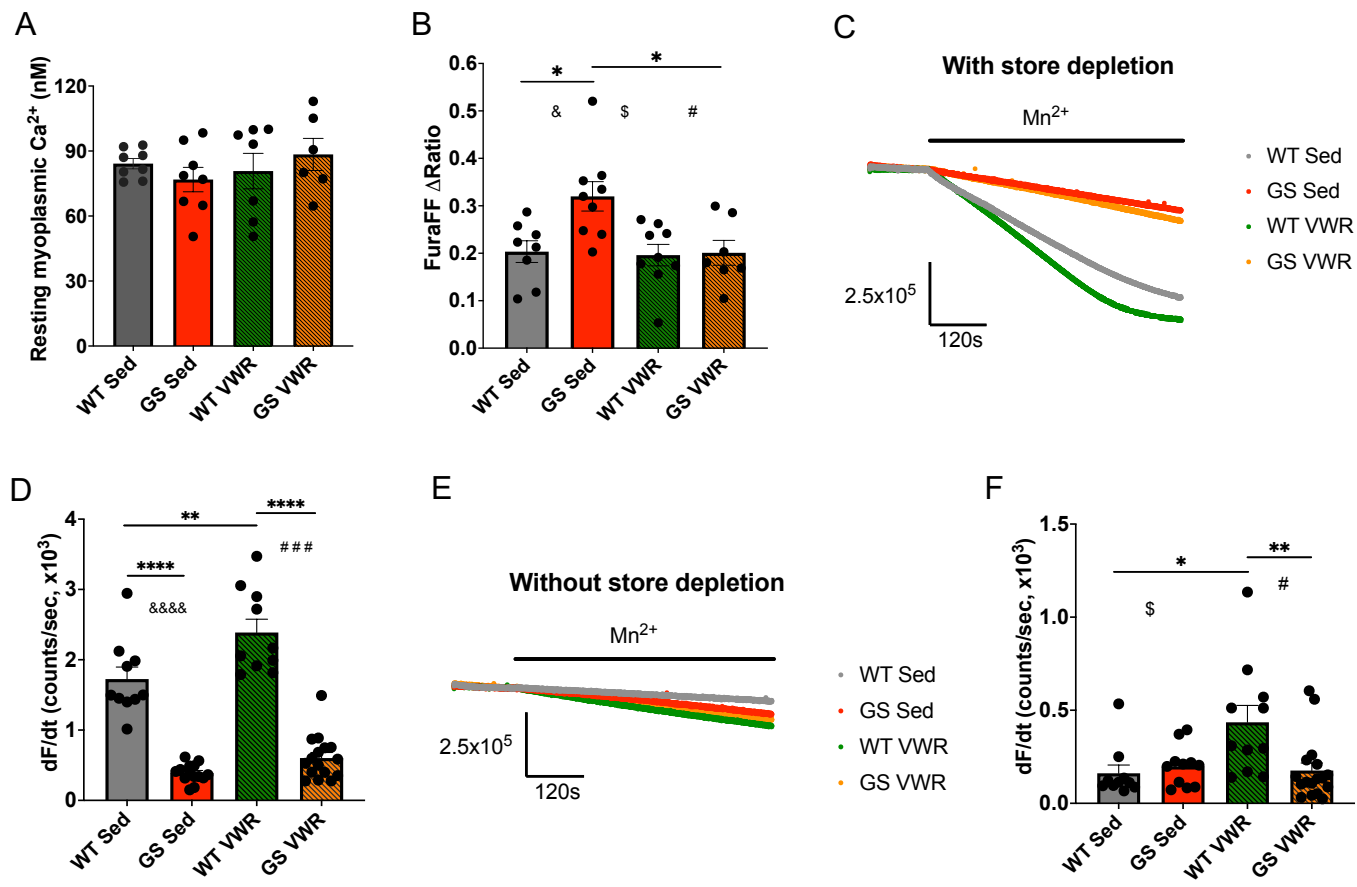

Supplement: Supplementary file 1 [file cells-14-01383-s001.zip › GS VWR Figures 20250821 Fig2.pdf]

## EDL

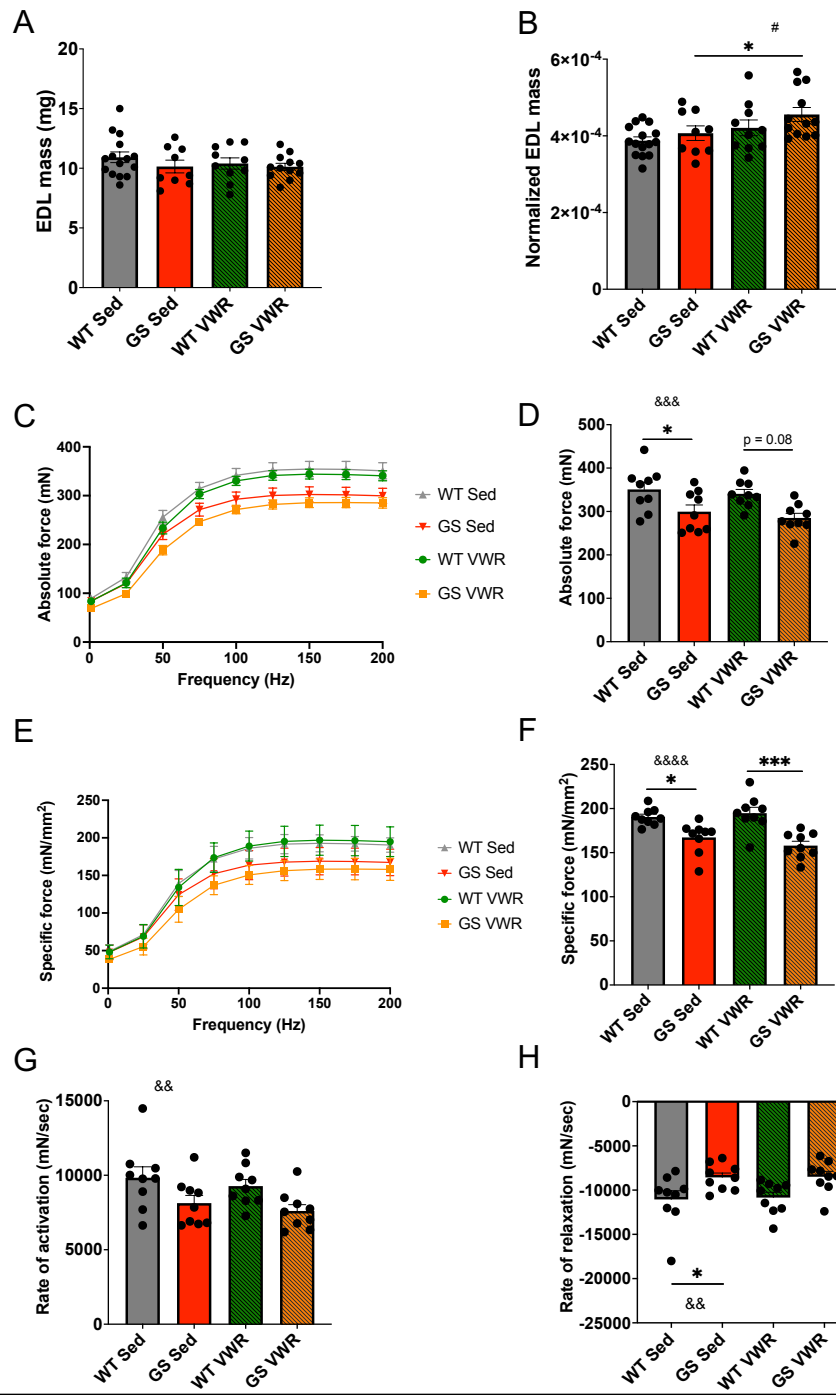

## SOLEUS

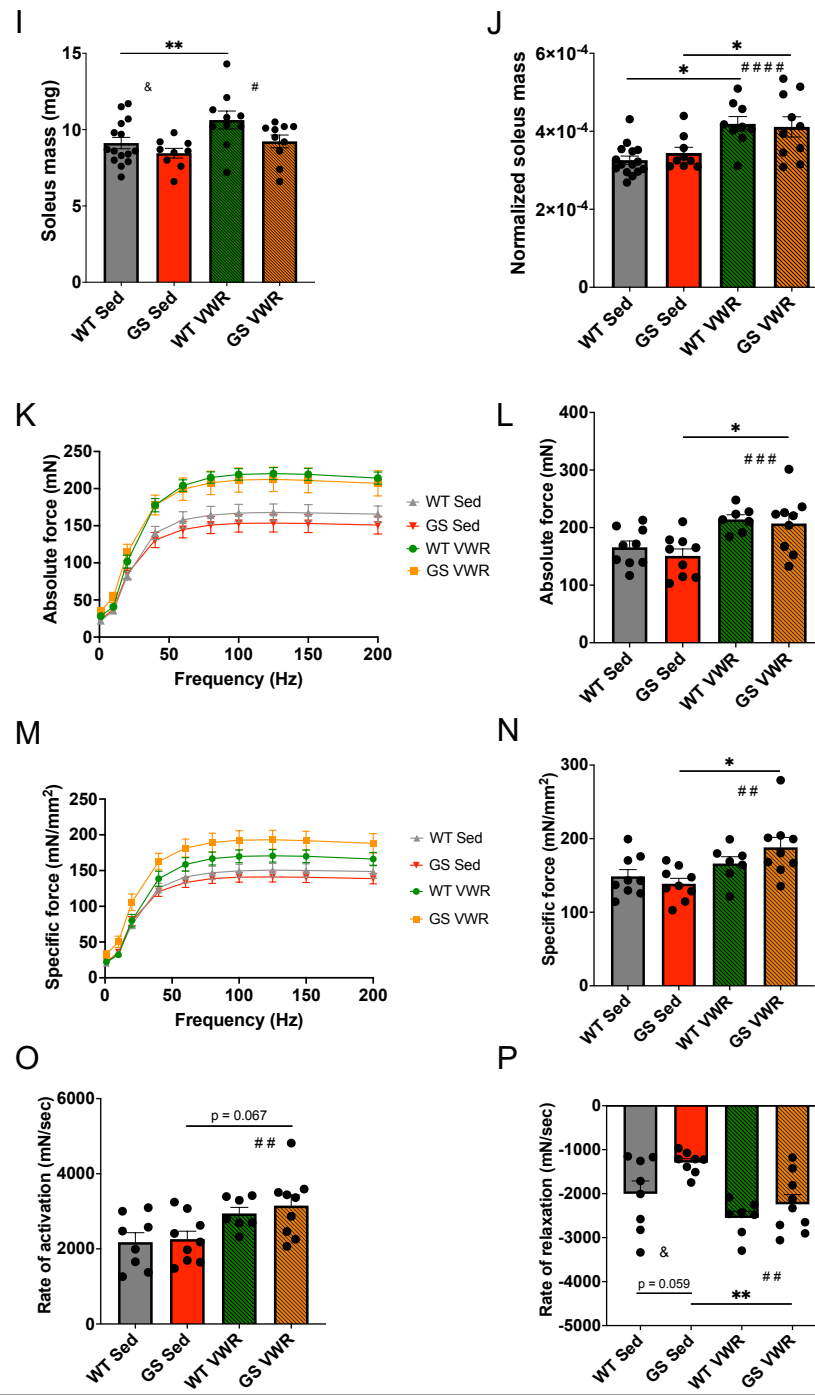

Fig. 3

Supplement: Supplementary file 1 [file cells-14-01383-s001.zip › GS VWR Figures 20250821 Fig3.pdf]

Fig. 5

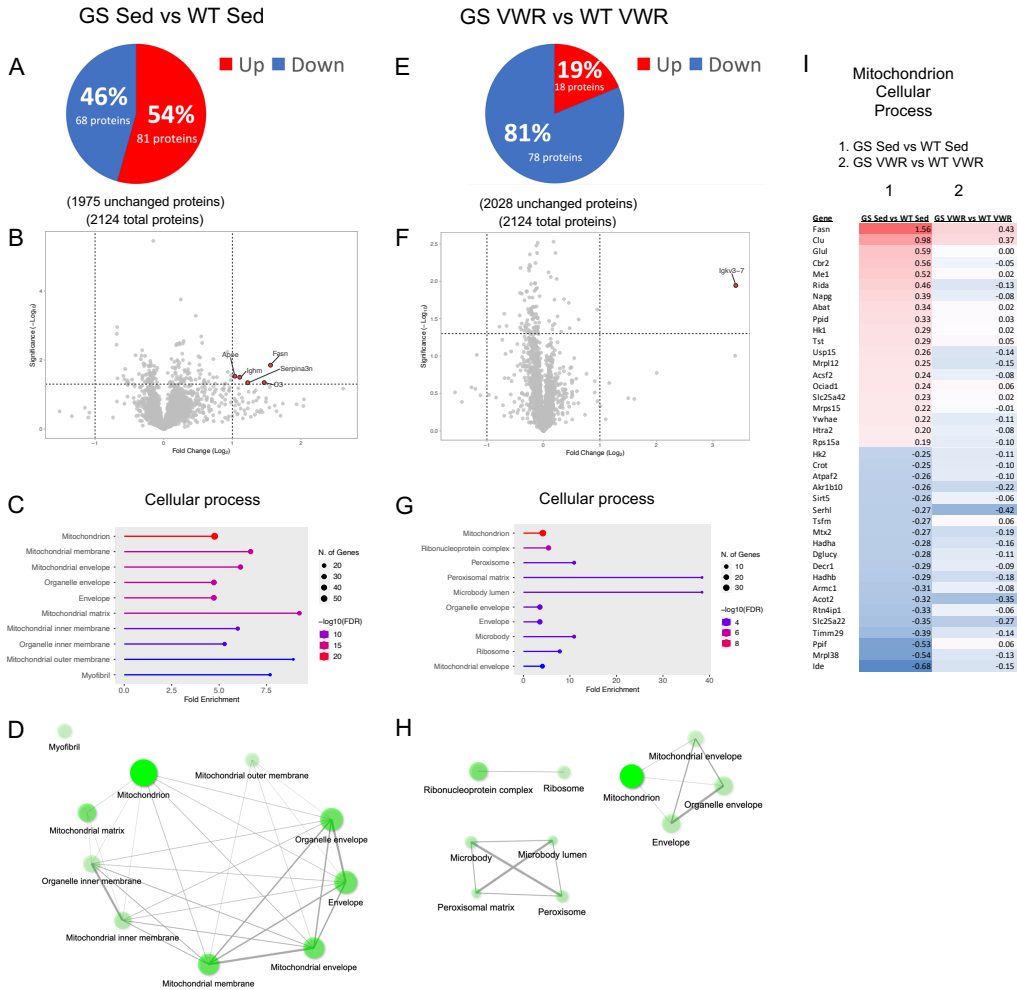

Supplement: Supplementary file 1 [file cells-14-01383-s001.zip › GS VWR Figures 20250821 Fig5.pdf]

Fig. 6

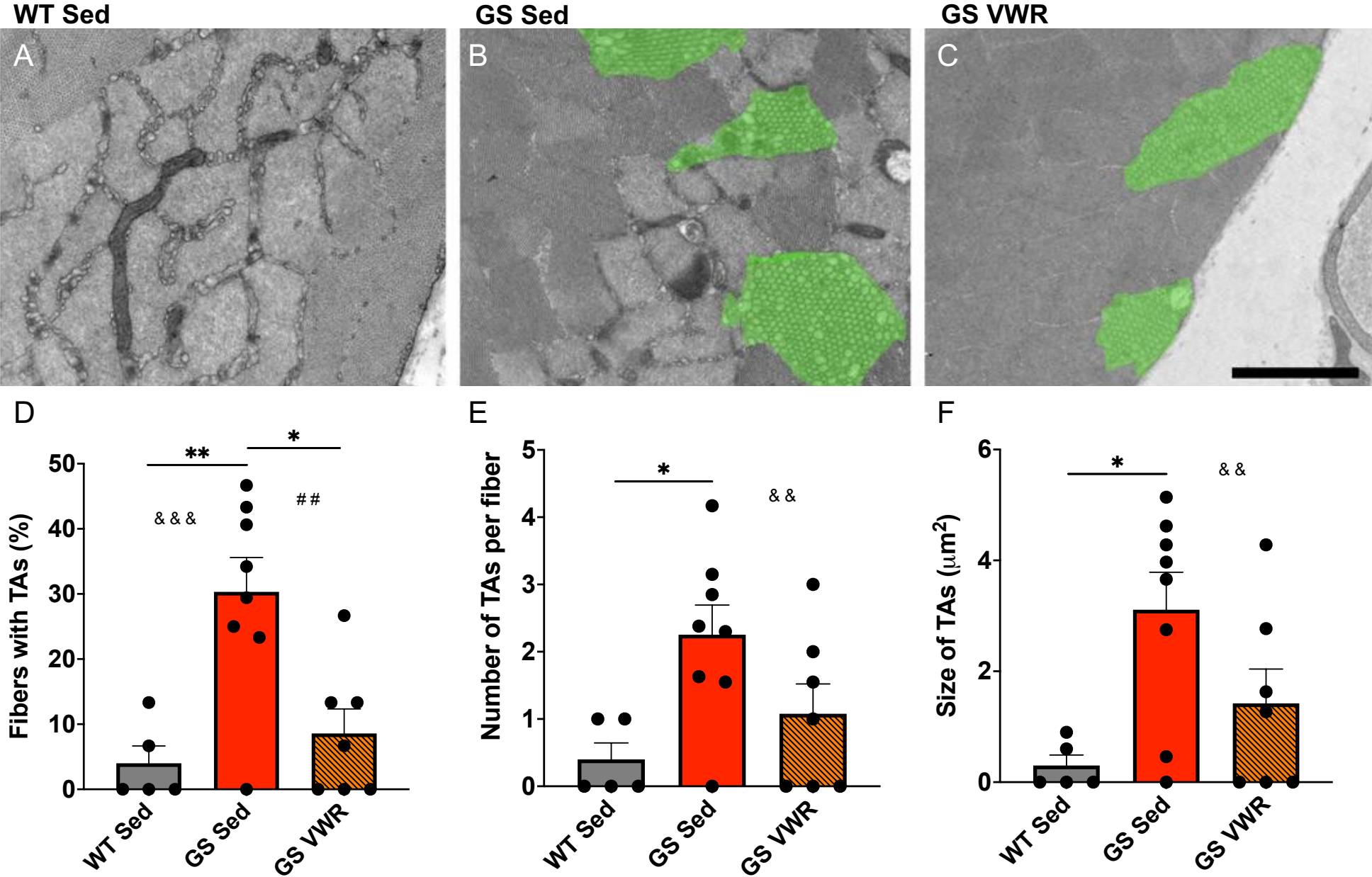

Supplement: Supplementary file 1 [file cells-14-01383-s001.zip › GS VWR Figures 20250821 Fig6.pdf]

Fig. 7

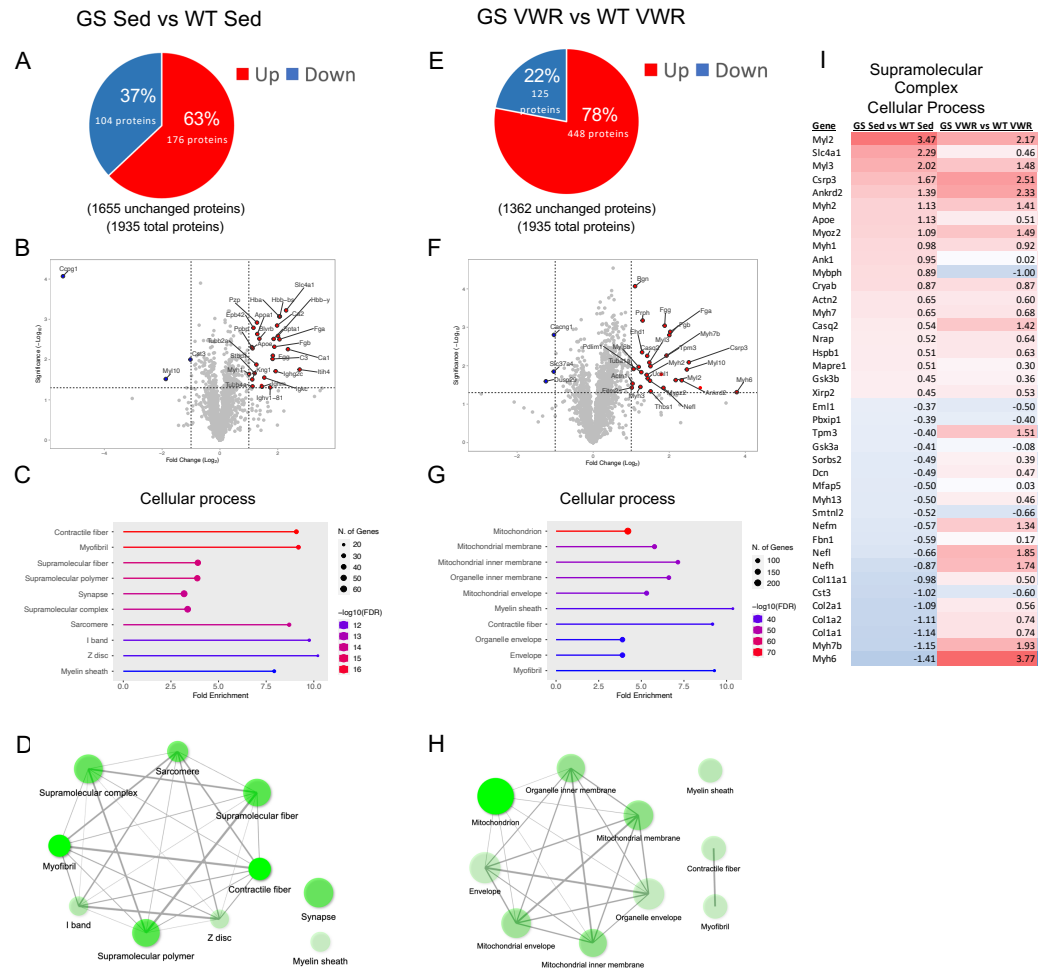

Supplement: Supplementary file 1 [file cells-14-01383-s001.zip › GS VWR Figures 20250821 Fig7.pdf]
