# Supplementary material for: Voluntary Wheel Running Mitigates Disease in an Orai1 Gain-of-Function Mouse Model of Tubular Aggregate Myopathy
Source: Cells. 2025 Sep 4;14(17):1383. doi: 10.3390/cells14171383 (PMC12427812; doi:10.3390/cells14171383)
Supplement: Supplementary file 1 [file GS VWR Figures 20250821 Fig4.pdf › GS VWR Figures 20250821 Fig4.pdf]

Fig 4.

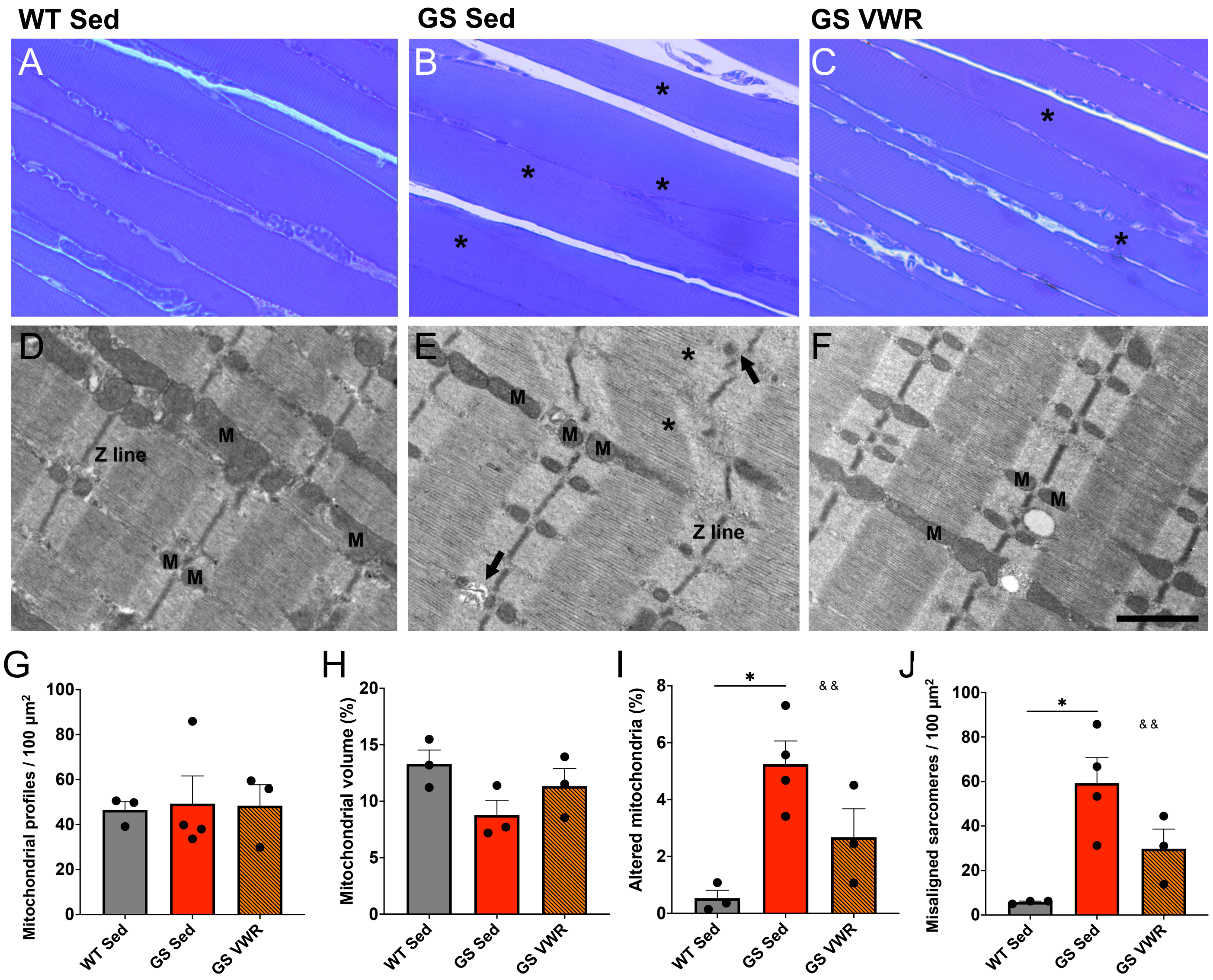

WT Sed
GS Sed
GS VWR
A
B
C
D
E
F
Z line
M
G
H
I
J
Mitochondrial profiles / 100 µm²
Mitochondrial volume (%)
Altered mitochondria (%)
Misaligned sarcomeres / 100 µm²
WT Sed
GS Sed
GS VWR
&&
